# Multinuclear NMR Measurements and DFT Calculations for Capecitabine Tautomeric Form Assignment in a Solution

**DOI:** 10.3390/molecules23010161

**Published:** 2018-01-13

**Authors:** Piotr Cmoch, Piotr Krzeczyński, Andrzej Leś

**Affiliations:** 1 NMR laboratory, Institute of Organic Chemistry Polish Academy of Sciences, Kasprzaka 44/52, 01-224 Warsaw, Poland; 2 Department of Chemistry, Pharmaceutical Research Institute, Rydygiera 8, 01-793 Warsaw, Poland; p.krzeczynski@ifarm.eu (P.K.); a.les@ifarm.eu (A.L.); 3 Faculty of Chemistry, University of Warsaw, Pasteura 1, 02-093 Warsaw, Poland

**Keywords:** capecitabine, tautomers, proton exchange, ^1^H-, ^13^C-, ^15^N-NMR, DFT

## Abstract

The molecular structure of capecitabine (a widely applied prodrug of 5-fluorouracil) was studied by multinuclear NMR measurements and DFT quantum mechanical calculations. One or two tautomeric forms in a solution were detected depending on the solvent used. In the organic solvents, a mixture of two forms of capecitabine was observed: carbamate and imine tautomers. In the aqueous solution, only the carbamate form was found. The methylation of capecitabine yields mainly two products in different proportions: *N*^3^-methylcapecitabine and *N*^7^-methylcapecitabine. The protonation of capecitabine in organic solvents with perchloric acid occurs at the N3 nitrogen atom. DFT calculations strongly support the results coming from the analysis of the NMR spectra.

## 1. Introduction

**Figure 1:**
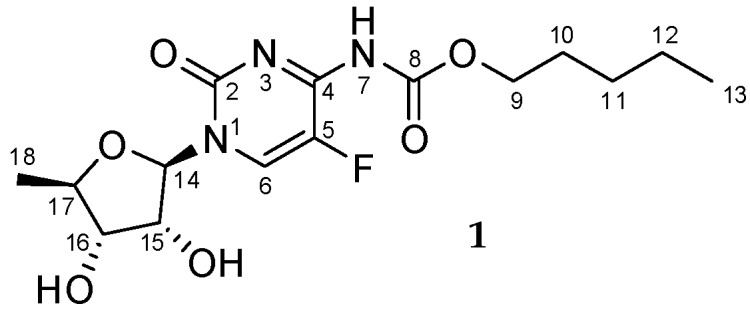
Notation of capecitabine **1**, the most frequently encountered in literature. The atom numbering used throughout this paper is shown.

Capecitabine **1** refers to pentyl (1-((2*R*,3*R*,4*S*,5*R*)-3,4-dihydroxy-5-methyltetrahydro- furan-2-yl)-5-fluoro-2-oxo-1,2-dihydropyrimidin-4-yl)carbamate, also known as Xeloda^®^ (by Roche) (Figure 1), and was first synthesized by Miwa et al. in 1998 [1]. Since then, a vast body of work has been published (over 12,000 citations according to the SciFinder—Chemical Abstracts Service) because capecitabine alone is an object of different complex studies and in combination with other active substances it is used in the treatment of breast, collateral, pancreatic, and other types of cancer [2,3,4,5]. The main idea that accompanied designing this chemotherapeutic substance commonly used in medicine was to improve the selectivity and bioavailability of its parent compound, 5-fluorouracil (5-FU). The 5-FU was designed as an oncological drug, which should selectively disrupt DNA replication in tumor cells. In vivo, capecitabine undergoes transformation to 5-fluorouracil in the course of enzymatic processes during which, as the first step, the carbamate side chain is hydrolyzed by liver carboxyesterase to form 5′-deoxy-5-fluorocytidine. The next steps take place in the liver as well as in tumor cells and rely on the action of cytidine deaminase leading to 5′-deoxy-5-fluorouridine, and further on capecitabine is released as a result of the thymidine phosphorylase enzymatic reaction in tumors [2,3].

**Figure 2:**
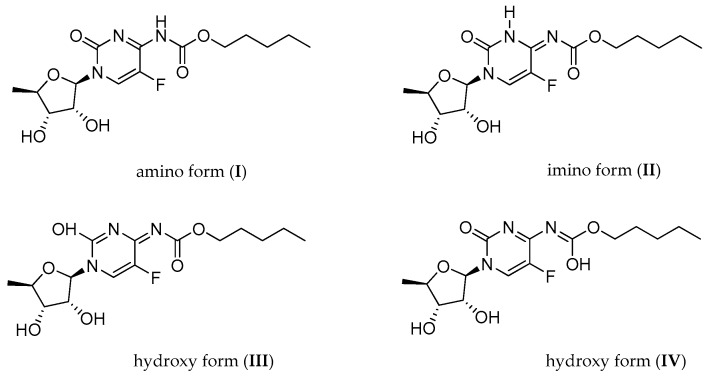
Possible tautomeric forms of capecitabine: **I**: carbamate-oxo, **II**: imine-oxo, **III**: imine- hydroxy, **IV**: enol-oxo.

Although use of capecitabine is widely described, especially in pharmacological and medical literature, a proper and correct notation of its molecular structure is still a matter of debate. The literature concerning capecitabine structure is rather poorly documented and only a few papers dealing with its synthesis are accessible [6,7,8,9,10,11,12,13,14,15,16]. From these previous papers presenting the synthesis of **1** and its ^1^H-NMR spectra, it appears that the organic solutions of capecitabine undergo a dynamic process. This is manifested by strong to medium NMR signal broadening and is probably related to the possible exchange of the NH protons between N/O atoms in the tautomeric forms presented in Figure 2.

Due to scarce literature, the data concerning capecitabine structure are incomplete and contradictory. All authors of the earlier papers mentioned the broadening of the ^1^H-NMR signals, but only a few papers presented a proper ^1^H-NMR spectrum (containing both exchangeable ^1^H-NMR signals) [6,7,10]. In others, only one “down-field” ^1^H-NMR signal was mentioned. Only three papers gave full ^13^C-NMR data of **1** as a single tautomeric form (**I**) [9,10,15] and even in two of them the ^13^C-NMR chemical shifts were assigned to the capecitabine structure (**I**) [9,10]. This assignment is rather strange/uncertain because in [10] the same author presented the original strongly broadened ^13^C-NMR spectrum in a DMSO solution. From a brief analysis of these ^13^C-NMR spectra, it is apparent that this dynamic behavior of capecitabine depends strongly on the solvent used.

**Figure 3:**
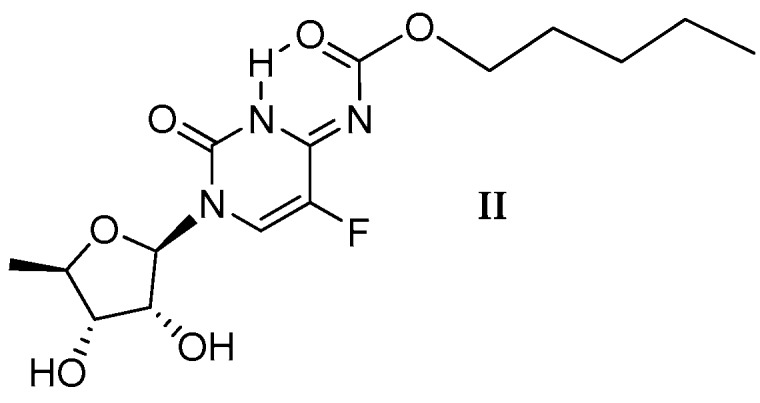
The preferred structure of imine tautomer **II** with the -C(=O)-O-C_5_H_11_ chain rotated around the C4-N7 bond by 180°, according to the X-ray measurements. An intramolecular N3-H⋯O(C8) hydrogen bond appears.

In 2009 Rohliček and co-workers [11], using synchrotron radiation at 293 K, reported the crystal structure of capecitabine **1** from the powder diffraction data as structure **1** (Figure 1). A few years later, our group [14] corrected the structure of capecitabine by presenting the X-ray data and postulated the preferred structure of capecitabine as an imine tautomer **II** with the -C(=O)-O-C_5_H_11_ chain rotated around the C4-N7 bond by 180° (Figure 3).

In 2016, Rohliček and co-workers [16] presented a correction to the previously published data, which is now in agreement with the data of Malińska et al. [14]. The authors of the crystallographic paper [14] also collected multinuclear MR data for **1** (in a solution and in the solid state) but no conclusions relating to its structure, apart from the “dynamic behavior of capecitabine”, were drawn. The ^13^C/^15^N CP-MAS spectra [14] presented in this paper contain the proper number of ^13^C and ^15^N signals corresponding to the structure of **1** but their positions in these spectra do not explain the structure/tautomeric form existing in the solid state. Based on the ^13^C and ^15^N CP-MAS chemical shifts only, it is rather difficult to predict if capecitabine exists in the solid state as a single tautomeric form or rather as a mixture of possible tautomers (Figure 2) for which the values of the ^13^C and ^15^N chemical shifts could be averaged.

The above information on capecitabine **1** and our interest in its behavior prompted us to clarify if a dynamic equilibrium exists in a solution of organic and non-organic solvents and what tautomeric forms are present there. To expand the knowledge about the capecitabine equilibrium, we decided to undertake temperature NMR measurements in different solvents. Additionally, methylated capecitabine isomers as model compounds were prepared for comparison purposes and their full NMR characteristics are presented. Finally, our goal was also to verify the postulate of Malińska and co-authors [14] about the need to change the notation of the structural formula found commonly in literature. In order to support our findings, we have also made use of the results of DFT quantum mechanical calculations.

## 2. Results and Discussion

As signaled above, we are now going to discuss shortly the CP-MAS results uncommented on in the previous paper [14]. The single sets of signals in ^13^C/^15^N CP-MAS spectra were detected for capecitabine in the solid state. Both these sets confirm the existence of capecitabine in one of the possible tautomeric forms (probably involved in the hydrogen bonds with another/partner tautomer), but which one can only be deduced on the basis of X-ray measurements. The ^13^C-NMR chemical shifts in the solid state for **1** are typical for this kind of compound and a decision on how their values correlate with a specific structure is completely unreasonable. The results of the ^15^N CP-MAS experiment provide better insight into the capecitabine structure. The ^15^N-NMR spectrum of **1** in the solid state taken at 298 K differs slightly from the one taken at 100 K and contains the following signals: δ = −241.9, −236.4 (NH), and −211.1 ppm [14]. We postulate that all three nitrogen signals have to originate from a single tautomer (**I**, **II**, **III** or **IV** (Figure 2)) involved in strong intermolecular hydrogen bonds with the partner molecule. The comparison of ^15^N chemical shifts in the solid state for **1** with the corresponding chemical shifts measured for cytidine **4** and 5′-deoxy-5-fluorocytidine **5** (present work) in DMSO (Figure S1, Supplementary Material), as well as cytidine 5′-monophosphate in water [17], leads to the suggestion that the presence of more than one tautomeric form of capecitabine in a solution should be expected.

To simplify the picture of this fairly complex equilibrium, we have decided to perform some fundamental calculations. Based on the results of the quantum mechanical estimations for all possible forms **I**–**IV**, we have assumed that hydroxy tautomer forms **III** and **IV** can be excluded. Their internal energies are above 100 kJ/mol over the lowest internal energy estimated for tautomer **II** (details in Table S1, Supplementary Material). Thus, further on in the paper, we will focus on the study of **I** and **II** tautomers only (in a solution) and the verification of the validity of this hypothesis will also be confirmed.

### 2.1. Multinuclear MR Measurements in the DMSO and THF Solutions

#### 2.1.1. Temperature 298 K: Tautomers **A** and **B** Are Detected

In the present ^1^H-NMR spectrum of capecitabine in the aprotic DMSO, two broad down-field signals (with different integrals) at δ = 10.52 and 11.68 ppm were observed (Table 1) (see also Figure S2). The ^1^H-NMR signal at δ = 10.52 ppm was also reported in the literature. It is interesting that the second signal at δ = 11.68 ppm was presented only in Hiyrianna’s Ph.D. thesis [10] and two patents [6,7]. The appearance of two low-field signals in the spectrum may originate from two tautomeric forms (at this point of the unknown structure) tentatively labeled **A** and **B**. Two forms of capecitabine are also observable in the ^19^F-NMR spectrum taken in the DMSO solution (Figure S3), where two relatively broad signals at δ = −159.8 ppm (form **A**) and −163.2 ppm (form **B**) with the integrals ratio 68:32 (Table 1), respectively, were recorded.

The presence of two tautomers in the DMSO solution of capecitabine at room temperature can also be deduced from the ^13^C-NMR spectrum. The overnight-accumulated ^13^C-NMR spectrum of **1** reveals more details than the spectra formerly published [9,10,15] and strongly shows broad signals (Table 1, also Figure S4, half-height width Δν_1/2_ is ca. 60–100 Hz, especially in the down-field range) originating from the pyrimidinone and carbamate molecular parts. The occurrence of two tautomers manifests as the presence of doubled signals (with different abundance) in the down-field area. Taking into account the predominance of form **A** (as it was assumed based on the ^1^H/^19^F-NMR spectra), some ^13^C-NMR signals can be assigned (based on different intensity) to the anomeric carbons of the ribofuranose ring at 89.6 ppm (form **B**) and 91.1 ppm (form **A**) and to the C6-H carbons of the pyrimidinone ring at 124.7 ppm (form **B**) and 129.7 ppm (form **A**). A tentative assignment of the rest of ^13^C-NMR signals is shown in Table 1. At this stage of the analysis, the identification of both tautomeric forms is still impossible due to the strong dynamic exchange process (and the associated broadening of the NMR signals) at room temperature. All these observations forced us to change the solvent and modify the temperature for multinuclear MR measurements.

The ^1^H- and ^19^F-NMR spectra of capecitabine at 298 K in THF showed a similar equilibrium pattern as in the DMSO solution. Surprisingly, contrary to the DMSO solution, both presumed forms **A** and **B** were almost equally populated in THF (Table 1).

#### 2.1.2. Low-Temperature ^1^H, ^19^F Spectra: Tautomer **A** Becomes More Abundant

In order to determine the tautomeric forms existing in the THF solution of capecitabine **1**, a series of temperature measurements were performed. Gradual temperature lowering causes considerable changes in Δν_1/2_ of both ^1^H/^19^F-NMR signals (Table 1). At 218 K the signals of exchangeable protons/fluorine atoms of both individuals (forms **A** and **B**) become narrow enough (10 to 20-fold for ^1^H, see: Figure S5 and 4 to 8-fold for ^19^F, see: Figure S6) that more advanced 2D NMR experiments can be performed.

Although the ^1^H/^19^F signals become sharper, the proton exchange still occurs and the corresponding NMR spectra show “dynamic” effects. Clear evidence of this is the results of low-temperature 2D NOESY/ROESY experiments in which both hydrogen nuclei (signals related to the NH protons at ca. 10.2 ppm and 11.9 ppm) strongly interact giving cross-peaks. A similar observation has been made for both aromatic protons (at δ = 7.86 ppm and 7.96 ppm).

The data presented in Table 1 show that the position of the tautomeric equilibrium in the capecitabine solution essentially depends on the temperature and solvent used. Form **A** (at 10.52 ppm and −159.8 ppm in ^1^H and ^19^F spectra, respectively) predominates in DMSO at room temperature, whereas both forms **A** and **B** exist in comparable amounts in the THF solution. Lowering the temperature slows the exchange in THF and the equilibrium is shifted towards the predominant form **A** (at 218 K the ratios of **A**:**B** are 71.7:28.3 and 70.0:30.0 based on the ^1^H and ^19^F integrals, respectively). A temperature decrease to 218 K in THF reveals the appearance of presumably the third form (a small peak visible in the ^19^F-NMR spectrum at about −162.2 ppm, Figure S6) but its integral is too small to be characterized by NMR. Therefore, this unknown form will be omitted in further considerations.

#### 2.1.3. Methyl Derivatives and Identification of **A**/**B** Tautomers

**Figure 4:**
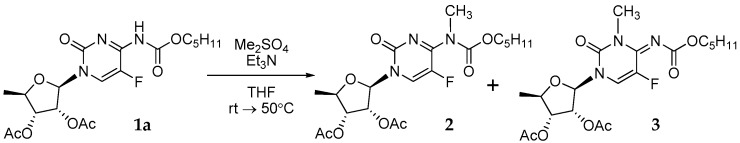
The isomeric *N*-methyl substituted acetyl protected capecitabine **2** and **3**.

In order to unambiguously identify the **A** and **B** tautomers, we decided to perform the methylation of acetylated capecitabine **1a**. After the rough purification, a crude mixture was obtained. The ^1^H-NMR spectrum (in DMSO-*d*_6_, 298 K) revealed two individuals, **2** and **3** (Figure 4), with the integral ratio 2.26:1.00 of the aromatic proton (H at C6) at δ = 8.04 and 8.41 ppm, respectively.

Which of the two isomers **2** and **3** in THF is more abundant will be demonstrated below. Details of the NMR characteristics for **2** and **3** in THF are presented in Table 2.

The *N*^7^-methyl **2** and *N*^3^-methyl **3** derivatives correspond to the fixed tautomeric forms of capecitabine (forms **I** and **II**). In order to correctly identify each newly synthesized isomeric compound **2** and **3**, the reaction mixture after methylation was subjected to a chromatographic separation. Furthermore, various 1D and 2D ^1^H/^13^C/^19^F/^15^N measurements for both methylated compounds formed the basis for the unequivocal identification and description of the isomers. We used the results of the ^1^H-^15^N g-HMBC, as well as the ^1^H-^13^C g-HMBC experiments, together with the analysis of the ^n^*J*(^13^C-^19^F) couplings to distinguish both model compounds **2** and **3**.

The sets of ^15^N-NMR signals for compounds **2** and **3** are extremely important because they correspond to the well-defined **A**/**B** (**I**/**II**) forms of capecitabine. To fully discriminate methyl derivatives **2** and **3**, a careful analysis of the cross-peaks coming from the ^1^H-^15^N long-range correlations is needed first. The identification of the N1 atom (bounded to the ribofuranose ring) and N3/N7 atoms that are methylated in the **2**/**3** forms is the easiest task. Strong correlations of the ribofuranose ring protons H14 (δ = 5.87 ppm)/H15 (δ = 5.46 ppm) and H6 pyrimidine proton at δ = 7.91 ppm in **2** are observed for nitrogen at δ = −226.2 ppm, relating this ^15^N-NMR chemical shift to the N1 nucleus. A similar type of ^1^H-^15^N correlations pointing to the N1 nucleus at δ = −255.9 ppm can be noticed for **3** (Table 2).

For both methyl groups at δ = 3.29 ppm (**2**) and 3.27 ppm (**3**), respective correlations with the signals at δ = −276.2 ppm and −245.6 ppm allow assigning of both N7 and N3 signals, respectively. The remaining correlations for both methyl derivatives of the protected capecitabine are −123.9 ppm and −151.7 ppm for N3/N7 of **2** and **3**, respectively. Therefore, a full set of ^15^N-NMR signals for **2** is −276.2 ppm (N7), −226.2 ppm (N1), and −123.9 ppm (N3); whereas for **3** it is −255.9 ppm (N1), −245.6 ppm (N3), and −151.7 ppm (N7) (see Table 2). In order to identify at which nitrogen atom the methylation occurs, further tedious examinations of other 2D ^1^H-^13^C-NMR spectra are necessary. The position of the ^1^H-^13^C long-range correlation cross-peaks, values of the ^n^*J*(^13^C-^19^F) couplings together with the information extracted from the ^1^H-^13^C g-HSQC experiments for **2** and **3**, allow confirming, without any doubt, the structure of both *N*-methylated capecitabine derivatives. For compound **2** the signals 153.0 ppm, 159.2 ppm [12.1 Hz], 140.5 ppm [243.9 Hz], and 154.5 ppm can be assigned to C2, C4, C5, and C8, respectively. Similarly, for **3** the respective signals are at 149.6 ppm, 145.6 ppm [26.0 Hz], 139.3 ppm [229.6 Hz], and 160.4 ppm. Additionally, the ^13^C-NMR chemical shifts for C2 and C6 carbons can be recognized and confirmed using ^1^H-^13^C long-range correlations with the ribofuranose protons H14. The correct identification of C2/C8 atoms is a prerequisite for the proper distinction between both isomeric methyl derivatives.

The position of the methyl substitution can be determined based on the ^1^H-^13^C long-range correlations of the methyl group at δ = 3.29 ppm (**2**) showing cross-peaks with two carbons at δ = 154.5 and 159.2 ppm and the second methyl group at δ = 3.27 ppm (**3**), where similar correlations indicate signals at δ = 149.6 ppm and 145.6 ppm. Based on the abovementioned assignments of the ^13^C-NMR signals for both methylated compounds, we can conclude that compound **2** corresponds to the *N*^7^-methyl isomer (analog of form **I** of capecitabine) and **3** denotes the *N*^3^-methyl isomer (analog of form **II** of capecitabine). The methylation of capecitabine **1** gave two methylated capecitabines, wherein the *N*^3^-methyl derivative (**3**) was more populated than the *N*^7^-methyl (**2**) derivative. It means that the nitrogen atom N3 is more susceptible to methylation.

Displacement of the methyl from N7 to N3 (**2** → **3**) causes significant changes in ^13^C/^15^N chemical shifts, as well as *J*(C-F) couplings. In the case of the ^15^N resonance, the most important is a strong shielding increase by over 120 ppm for the N3 nucleus and simultaneously a similar deshielding effect for N7, as well as the N1 shielding increase by ca. 30 ppm. The transfer of the methyl group from N7 to N3 involves changes of all pyrimidinone proton/carbon/fluorine nuclei H6, C2, C4, C5, and C6. Relatively strong shielding increases are observed in the case of the nuclei C4 (14 ppm), C6 (9 ppm), and F (6 ppm), whereas for C8 an opposite effect by ca. 6 ppm is noticed. Attention should also be paid to the values of the ^n^*J*(^13^C-^19^F) couplings. Transition from the *N*^7^-methyl substituted capecitabine (**2**) to the *N*^3^- derivative (**3**) causes an increase of ^2^*J*(^13^C-^19^F) by ca. 14 Hz at C4 and a decrease of ^1^*J*(^13^C-^19^F) by the same value at C5 (Table 2). Changes in the NMR parameters mentioned above may be helpful in the process of identifying the tautomeric forms of capecitabine and similar compounds, also in other conditions.

#### 2.1.4. Capecitabine in THF: Form **I** Dominates over Form **II**

To identify tautomeric forms of capecitabine existing in the THF-*d*_8_ solvent at 218 K, the ^1^H-^15^N g-HSQC experiment was performed. Two correlation cross-peaks were observed: form **A** (more widespread) δ = 10.08 ppm (1H)/−268.9 ppm (^15^N at N7) and form **B** 11.88 ppm (^1^H)/−236.5 ppm (^15^N at N3). The values of the ^15^N-NMR chemical shifts for both N-H nitrogens are similar to those obtained for the *N*-methyl derivatives (**A** similar to **2** and **B** similar to **3**) and differ by less than 10 ppm. This effect of the methyl/proton replacement is known and typical for a nitrogen atom of this type [17,18]. A careful analysis of the 2D ^1^H-^15^N HMBC experiment distinctly indicates sets of ^15^N signals for each tautomeric form. The full assignment of the ^15^N signals for **A** and **B** is based on the method presented above for the methyl derivatives of capecitabine. For the more populated form **A**, the ^15^N-NMR chemical shifts are as follows: −268.9 ppm (N7), −221.7 ppm (N1), and −139.3 ppm (N3); whereas for form **B**, only two signals can be noticed: −244.6 and −236.5 ppm (N3) (Table 2). The assignment of the ^15^N-NMR signal at δ = −244.6 ppm for **B** is rather uncertain because the only cross-peak in which it takes place is related to N-H proton at δ = 11.88 ppm. This proton can couple with both nitrogen atoms (N1 and N7) across three bonds and the conclusion of which ^3^*J*(^1^H-^15^N) is more effective (giving a cross-peak) remains somewhat unclear. We can only assume that the ^15^N chemical shift for N1 in the second form (**B**) should be close to the value for the N1 nucleus (δ = −255.9 ppm) noticed for **3**. The comparison of the ^15^N chemical shifts obtained for **A** with those for **2** (Table 2), as well as for other structural analogs of capecitabine (i.e., cytidine (also in [19,20]) and 5′-deoxy-5-fluorocytidine in DMSO-*d*_6_ [19] (compounds **4** and **5**, Figure S1)) confirm that form **A** is carbamate **I.** The ^15^N data for form **B** are incomplete but likely related to the imine form **II**. This assignment is supported by the ^15^N signals extrapolated from the available experimental data using DFT calculations (−191.9 ppm).

Just as in the case of the ^15^N-NMR signal assignment, the use of the long-range correlations ^n^*J*(^1^H-^13^C) coming from the ^1^H-^13^C g-HMBC experiment allows an appropriate carbon nuclei to be identified and to assign ^13^C-NMR signals. The best assignment is presented in Table 2.

Similarly, as in the case of the protected capecitabine methyl derivatives **2** and **3**, the displacement of the proton from N3 (form **II**) to N7 (form **I**) causes a strong ^15^N shielding decrease by ca. 100 ppm for N3 and ca. 20 ppm for the N1 nucleus. The ^13^C shielding/deshielding effects observed in **II** and **I** are also of significant importance; however, they are less consistent than the effects observed for methyl derivatives. The C2, C6, and F are more deshielded by ca. 7 ppm, 3 ppm, and 2 ppm, respectively, after the displacement of the proton from the N3 to N7 atom. Simultaneously, a shielding increase is noticed for the C5 and C8 atoms of ca. 2 ppm and 13 ppm, respectively. The values of the ^1^*J*(^13^C-^19^F) for the C5 atom in both tautomeric forms (232.4 Hz (**II**) and 243.6 Hz (**I**)) (Table 2) remain almost unchanged (compared to the isomers **3** and **2**).

The comparison of the ^1^H-NMR and ^19^F-NMR integrals of the low-field signals (at 218 K) for both tautomeric forms of capecitabine (Table 1) makes it clear that a temperature decrease leads to the predominance of the carbamate form **I**. This conclusion is in contradiction with the predisposition of imine **II** to produce a more preferred form stabilized by the intramolecular hydrogen bond (Figure 3) and at the same time with the conclusion drawn based on the results coming from crystallographic studies [14]. Such behavior of capecitabine in the aprotic solution is rather unexpected.

#### 2.1.5. Capecitabine in the Aqueous Solution: Form **I** Is Exclusively Detected

The ^1^H-NMR spectrum of capecitabine in H_2_O/D_2_O is relatively sharp and does not present traces of dynamic behavior. Moreover, the number of ^1^H-NMR signals corresponds to the only one single form of the main compound, which is confirmed by the NMR spectra of the ^19^F and ^13^C nuclei. Based on the sole ^1^H spectrum, the specific tautomeric form of capecitabine **I**/**II** cannot be identified and the use of other NMR data arbitrates this question unambiguously. The comparison of the ^13^C-NMR chemical shifts, especially of the pyrimidinone and carbamate part, as well as ^n^*J*(^13^C-^19^F) measured for capecitabine in water, with those presented earlier for both forms of capecitabine at 218 K and both methyl derivatives **2** and **3** in THF at 298 K, strongly suggests the existence of the carbamate form **I** in water. Such a conclusion can be drawn because the ^13^C-NMR chemical shifts and values of ^n^*J*(^13^C-^19^F) couplings at C5 and C4 for capecitabine in H_2_O/D_2_O are very similar to those of form **A** (**I**) of capecitabine in THF, particularly to the model compound **2** (Table 2). Our efforts to measure the ^15^N-NMR spectrum for **1** in water ended only with a partial success. Namely, in the ^1^H-^15^N g-HMBC correlation spectrum, two weak cross-peaks were noticeable. The ^1^H signals at δ = 4.30 and 5.78 ppm (sugar part) correlate with only one ^15^N signal at δ = −225.0 ppm. This ^15^N-NMR chemical shift corresponds to nitrogen N1 and this value, after a comparison with other values for both tautomeric forms and the methyl representative of both tautomers (Table 2), clearly indicates the existence of capecitabine as the carbamate form **I** in water. A detailed assignment of the ^1^H/^13^C/^19^F and ^15^N-NMR signals is presented in Table 3. The form **I** of capecitabine should also be the one encountered in numerous studies on human liquids and organs [21,22,23] whose major content is water. At this stage of our studies, it should be mentioned that there is no reason to revise the notation of the capecitabine structure, as suggested previously [14].

#### 2.1.6. Capecitabine in an Acidic Medium: Form **I** Is Protonated at the N3 Atom

The next challenge was to investigate the capecitabine molecular structure in an acidic medium and attempt to describe how the addition of an acid to the solution in an aprotic solvent (THF) affects capecitabine **1**. In the ^1^H-NMR spectrum of an equimolar mixture of capecitabine and perchloric acid (at 283 K, immediately after mixing), a single set of signals, very similar to the proton spectrum of capecitabine in H_2_O/D_2_O, is noticeable. The comparison of the ^13^C-NMR spectrum for the acidic solution of capecitabine with the one registered in the aqueous solution indicates significant changes in the ^13^C-NMR chemical shifts, probably related to the protonation of the capecitabine nitrogen atom. The most important are as follows: strong shielding increase by ca. 11 ppm for C2 and 4 ppm for C4 and C5 carbons, respectively. The opposite effect (ca. 4 ppm) is observed for C6, whereas the ^13^C-NMR chemical shift for C8 remains almost unchanged. These effects essentially relate to the protonation of the nitrogen N3 atom, but the use of the ^15^N-NMR chemical shifts can confirm the process itself and the place where it occurs. Although at lower temperatures the ^1^H signals of both protons (one of capecitabine and the second from an acid) are not visible in the ^1^H-NMR spectrum due to a fast exchange, the values of the ^15^N-NMR chemical shifts can be obtained from the ^1^H-^15^N HMBC experiment. The ^15^N signal at δ = −221.0 ppm belongs to the N1 nucleus because cross-peaks at the positions of H14/H15 and H6 protons exist. Two remaining nitrogen signals at δ = −225.8 and −259.4 ppm coming from the cross-peaks of the H6 proton necessarily correspond to the N3 and N7 nuclei. It is not clear which ^15^N-NMR chemical shift corresponds to the given nitrogen atom but the comparison of the ^15^N chemical shifts of **I**/**II** (Table 2) with those acquired for capecitabine in acidic conditions and a commonly known effect of the nitrogen shielding increase by ca. 90–100 ppm after protonation allow for their reasonable assignment. The NMR data for protonated capecitabine in the THF solution is presented in Table 3.

### 2.2. Quantum Mechanical DFT Calculations

A complicated picture of experimental NMR data for capecitabine and its methyl congeners in various solutions at different temperatures prompted us to perform the quantum mechanical study on model systems in order to gain insight into the molecular basis of capecitabine tautomerism. Two factors were considered, namely the internal molecular stability and the intermolecular interactions with the solvent molecules. The internal molecular stability was analyzed for **I**–**IV** tautomeric forms of capecitabine. It appeared that form **II** becomes the lowest energy tautomer, then follows tautomer **I** of about 10 kJ/mol higher than **II** (see: Tables S1 and S2). As the result of the calculations, forms **III** and **IV** were rejected due to their highly unfavorable molecular energy (about 100 kJ/mol above the energy of **II**). The predicted prevalence of tautomer **II** corresponds to the capecitabine crystal structure [14] showing the presence of tautomer **II** only.

The effects of the intermolecular interactions on the ^1^H chemical shift were studied in capecitabine-DMSO and capecitabine-THF binary complexes, as well as in the ternary capecitabine-(H_2_O)_2_ complex. The solvent molecules were bound by hydrogen bonds of about 15–44 kJ/mol following the B3LYP/6-311G(2d,2p) calculations counterpoise corrected (see also Figures S7–S9 for details). It is likely that a strength of about 40 kJ/mol characterizes possible capecitabine dimers, which can interact similarly as in the solid state [14]. The effect of the solvent on the relative internal energy of **I** and **II** tautomers can also be estimated using the total electronic energy corrected with the contribution from the interaction with the solvent estimated with the use of the continuum solvent model (SMD). For the isolated (non-interacting) tautomers, form **II** (imino) prevails. Then, the prevalence of form **II** gradually diminishes in THF, approaching almost zero in DMSO. In the aqueous solution, form **I** becomes more favorable (see Table S4 for details).

We have also studied the solvent effect on the chemical shift and spin-spin coupling constants. It was predicted for example that the proton attached to the nitrogen atom and not involved in the hydrogen bond showed a chemical shift of about 7 ppm while with the hydrogen bond the chemical shift increased to 10 ppm (in tautomer **I**) or 12 ppm (in tautomer **II**) (Table S3). It is worth noticing that tautomer **II** already has an intramolecular hydrogen bond N3-H⋯O(C8). The present result may explain the appearance of 10.52 ppm and 11.68 ppm signals in the ^1^H-NMR spectrum of capecitabine in DMSO (Figure S2). A comparison of the DFT and experimental ^1^H, ^13^C, ^15^N, and ^19^F spectral parameters for capecitabine (form **I** and **II**) in DMSO, THF, and H_2_O solvents, as well as in the acidic solution in THF (modeled as capecitabine protonated form **I** encapsulated by THF), is shown in Tables S3–S11. A prediction of the NMR parameters by the corresponding DFT values becomes satisfactory and is characterized by the root-mean-squared-error below 3 ppm for ^1^H, ^13^C, and ^1^J(C5-F). A less satisfactory prediction was obtained for the ^15^N chemical shifts of about 10 ppm, which is actually acceptable for estimating certain unknown (unavailable experimentally) chemical shifts of the capecitabine nitrogen atoms. In general, one can say that in spite of the relatively advanced theoretical approach, there remains a visible discrepancy between the predicted quantum mechanical and experimental spectral parameters. In most cases, the discrepancy does not exceed 5% (see Table S5 for more details), allowing for the support of the tautomer assignment deduced from the NMR spectra. Surprisingly, the fluorine chemical shifts predicted by DFT (using different functionals and basis sets) show a reversed order of tautomers when compared to the experimental data, although the magnitude and separation of peaks originating from forms **I** and **II** are reasonable (Table S6). An attempt to elucidate this problem will be undertaken in the future.

Several reasons can be considered for the potential source of such a discrepancy: for example, the crude modeling of the capecitabine solution represented by a single molecule encapsulated by a continuous medium mimicking solvent molecules, limitations of the DFT approach to reflect the quantum nature of the interacting nuclei and electrons, as well as the medium-size Gaussian basis sets used in the approximation of the electron density within the DFT theory. A more prospective result can be obtained if one considers a correlation between experimental and theoretical data expressed in the form of a linear trend adopted for the selected series of the NMR parameters (Tables S5 and S9–S11). With the use of such a linear relation, one can predict some of the lacking experimental results (c.f. results in Table 1, Table 2 and Table 3 presented in italics).

The present theoretically predicted chemical shifts and spin-spin coupling constants combined with the experimental counterparts suggest that both **I** and **II** forms of capecitabine can appear in non-aqueous solutions. The prevalence of a particular tautomeric form depends on the intra- and intermolecular interactions, including hydrogen bonds.

## 3. Materials and Methods

### 3.1. Synthesis of Capecitabine N^3^/N^7^-Methyl Derivatives **2** and **3** (Figure 4)

Step 1:

Place 2.00 g (6 mmol; 1.0 eq.) of 2′,3′-di-*O*-acetyl-5′-deoxy-5-fluorocytydine and 12 mL of dichloromethane in a 100 mL 3-neck round bottom flask equipped with a dropping funnel, reflux condenser, and magnetic stirring bar. After solid dissolution, 0.7 mL (9 mmol; 1.5 eq.) of pyridine was added. Next, 1.1 mL of *n*-pentyl chloroformate was placed in the dropping funnel and added drop-by-drop to the vigorously stirred starting solution, while maintaining the reacting mixture under soft reflux. When the *n*-pentyl chloroformate addition was completed, the solution was stirred for 1 h and then cooled to room temperature, poured into the separation funnel, and washed with 10 mL of aq. CuSO_4_, 10 mL of brine, and 10 mL of water, then dried over MgSO_4_. The clear solution was evaporated twice from 5 mL of the toluene solution giving 2.55 g of **1** as a white solid.

Step 2:

The solid obtained in step 1 was dissolved in 5 mL of dichloroethane. To this clear, greenish solution 2 mL triethylamine followed by 1.4 mL dimethyl sulfate was added and the resulting solution was maintained at 50 °C overnight. After cooling to room temperature, the mixture was diluted with 20 mL dichloroethane, washed twice with 10 mL water, dried over MgSO_4_, and evaporated, yielding 2.25 g of the crude product. The separation using flash chromatography (CH_2_Cl_2_/MeOH 100:0 followed by 99.5:0.5 then 99:1) resulted in 0.20 g of the *N*^7^-methyl (**2**) and 0.59 g of the *N*^3^-methyl derivative (**3**).

### 3.2. NMR Spectroscopy

All NMR measurements were performed using Varian-NMR-vnmrs600 or Varian-NMR-vnmrs500 spectrometers (Varian, Palo Alto, CA, USA, at different temperatures and in various solvents) equipped with a PFG Auto XID (^1^H/^15^N-^31^P 5 mm) indirect probehead or a PFG Auto XDB (^1^H-^19^F/^15^N-^31^P 5 mm) direct probehead, respectively. Standard experimental conditions and standard Varian programs (ChemPack 4.1, Varian, Palo Alto, CA, USA) were used. To assign the structures, the following 1D and 2D experiments were employed: the ^1^H selective NOESY/ROESY, COSY, ^1^H-^13^C gradient selected HSQC (g-HSQC) and HMBC (g-HMBC) optimized for ^1^*J*(C-H) = 146 Hz and ^n^*J*(C-H) = 8 Hz. Additionally, the ^1^H-^15^N gradient selected HSQC and HMBC optimized for ^1^*J*(N-H) = 90 Hz and ^n^*J*(N-H) = 6 Hz and 12 Hz were used, respectively, to distinguish different types of the nitrogen atom in the molecules investigated.

The ^1^H and ^13^C-NMR spectral data are given relative to the TMS signal at 0.0 ppm or THF up-field signals at 1.73 and 25.3 ppm for ^1^H and ^13^C nuclei, respectively. Nitromethane (signal at 0.0 ppm) was used as the external standard for the ^15^N-NMR spectra. Depending on the solvent, the concentrations of all solutions used for the measurements were about 30–50 mg of the compound in 0.6 mL (or 0.19 ± 0.05 mol/L).

### 3.3. DFT Calculations

The quantum mechanical calculations were performed with the density functional theory DFT (B3LYP [24], wB97XD [25,26]) using the 6–31G(d,p), 6–311++G(2d,2p) and pcJ–1 [27] Gaussian basis sets. The starting geometry for the imine form **II** was chosen based on the recent crystal structure [14]. The carbamate tautomeric form was invented by the formal proton transfer from the *N*^3^ position at the pyrimidine ring towards the exocyclic N7 nitrogen and then re-optimized. The starting geometries of other structures were generated from these basic structures. The optimal geometries of all models were obtained following Berny’s optimization algorithm and confirmed with all positive harmonic frequencies. The NMR shieldings and spin-spin coupling constants were obtained with the NMR GIAO [28,29] method implemented in the Gaussian G09 (rev. B.01) suite of programs [30] (and references therein). The SMD continuum solvent model [31] was used as implemented in the Gaussian G09.

## 4. Conclusions

The multinuclear NMR data (chemical shifts and coupling constants) in combination with the results of a careful analysis of various 2D experiments for the methyl derivatives of capecitabine led to the identification and distinction of tautomeric forms of the main compound. Based on the full sets of the NMR parameters for each tautomer, the presence of two tautomeric forms of capecitabine was detected in the aprotic organic solvents (DMSO and THF). Two forms (**I** and **II**, Figure 2) in comparable amounts were observed in the aprotic THF solutions at room temperature, whereas in the more polar and vicious aprotic DMSO solution the carbamate form **I** was dominant. Lowering the temperature in THF increased the population of form **I**. The comparison of the NMR parameters for capecitabine and its methyl derivatives with those obtained for capecitabine in H_2_O/D_2_O shows that the predominant and only form in water is carbamate **I**. Moreover, the acidification of the aprotic THF solution containing capecitabine resulted in the protonation of capecitabine at the N3 pyrimidinone nitrogen atom of the carbamate form **I**. The comparison of the ^15^N-NMR chemical shifts for protonated capecitabine in the solution (δ = −221.0, −225.8 and −259.4 ppm) with those for capecitabine in the solid state presented earlier [14] (δ = −211.1, −236.4 and −241.9 ppm) showed distinctly that the participation of hydrogen bonding plays a key role in establishing the molecular structure in the solid state [14]. The issue of capecitabine’s correct notation still remains open but it seems that there is no need to change the notation of capecitabine used so far (**I**).

The experimental findings are supported by the quantum mechanical DFT calculations of the molecular energies, nuclear shielding constants, and spin-spin coupling constants. The theoretically predicted NMR parameters correlate reasonably well with the experimental counterparts. The estimation of certain unavailable experimental data is possible with the use of the linear regression.

## Figures and Tables

**Table 1:** The ^1^H/^19^F, ^13^C-NMR chemical shifts, ^1^H/^19^F Δν_1/2_ and integrals (of the down-field signals) for **A** and **B** forms of capecitabine at 298 K in DMSO and THF, as well as at 218 K in THF.

**^a^**	**Chemical Shift (ppm)**		**Ratio of the Integrals ^c^**		**Δν_1/2_ (Hz)**	
	**DMSO-*d*_6_**	**THF-*d*_8_**	**DMSO-*d*_6_**	**THF-*d*_8_**	**DMSO-*d*_6_**	**THF-*d*_8_**
^1^H	10.52 (**A**)	9.61 (**A**)	66.5	48.6	62	185
298 K	11.68 (**B**)	11.63 (**B**)	33.5	52.4	168	141
^1^H		10.21 (**A**)		71.7		5
218 K		11.88 (**B**)		28.3		11
^19^F	−163.3 (**B**)	−164.7 (**B**)	32.1	51.8	250	176
298 K	−159.8 (**A**)	−162.6 (**A**)	67.9	48.2	115	192
^19^F		−163.3 (**B**)		30.0		17
218 K		−161.5 (**A**)		70.0		64
**^b^**	**^13^C-NMR Chemical Shifts (ppm) DMSO-*d*_6_ at 298 K**					
	**Form A**			**Form B**		
C6	129.7			124.7		
C5	137.0 [244 Hz] ^d^			Not found, (*140.6*) [*224.3 Hz*] ^f^		
C2, C4, C8	151.2, 152.5, 153.7 ^e^			147.7, 161.0, not found ^e^		
C14	91.1			89.6		

^a^ The ^1^H-NMR chemical shifts for the down-field signals only; see also a complete ^1^H-NMR spectrum (Figure S2); ^b^ The ^13^C-NMR chemical shifts for pyrimidine/CO carbons; see also a complete ^13^C-NMR spectrum (Figure S4); ^c^ Ratio of the integrals normalized to 100% based on the spectra shown in Figures S2, S3, S5, and S6 in the Supplementary Material; ^d 1^*J*(^13^C-^19^F); ^e 13^C-NMR signals cannot be properly assigned; ^f^ In parentheses and in italics: values predicted with the use of the linear regression; details are given in Table S5.

**Table 2:**
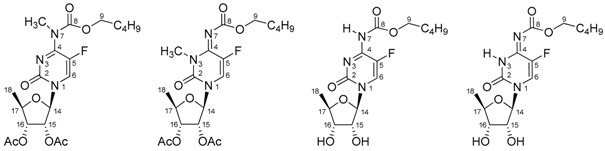
The ^1^H-, ^13^C-, ^19^F- and ^15^N-NMR chemical shifts in THF-*d*_8_ for the tautomeric forms of capecitabine **1** (at 218 K) and its methyl derivatives **2** and **3** (at 298 K).

	2	3	A = I	B = II
N1	−226.2	−255.9	−221.7	−244.6
C2	153.0	149.6	154.0 ^1^	147.4
N3	−123.9	−245.6	−139.3	−236.5
C4	159.2 [12.1 Hz] ^2^	145.6 [26.0 Hz] ^2^	154.4 1 [11.8 Hz] ^2^	154.0 ^1,3^
C5	140.5 [243.9 Hz] ^2^	139.3 [229.6 Hz] ^2^	137.9 [243.6 Hz] ^2^	140.3 [232.4 Hz] ^2^
F	−154.5	−161.0	−161.5	−163.3
H6/C6	7.91/130.3 [36.2 Hz] ^2^	7.52/121.3 [36.0 Hz] ^2^	7.96/129.7 [34.0 Hz] ^2^	7.86/126.5 [34.5 Hz] ^2^
N7	−276.2	−151.7	−268.9	(−*197.8*) ^4^
H7/H3	-	-	10.08	11.88
CH3	3.29/34.7	3.27/30.4	-	-
C8	154.5	160.4	151.7	164.7
H9/C9	4.17/67.8	4.05/66.4	4.11/66.5	4.05/66.3
H10/C10	1.65/29.2	1.63/29.4	1.65/29.4	1.65/29.4
H11/C11	1.35/28.8	1.35/29.0	1.35/28.9	1.35/29.2
H12/C12	1.35/23.2	1.35/23.2	1.35/23.5	1.35/23.6
H13/C13	0.91/14.3	0.90/14.3	0.91/14.7	0.91/14.7
H14/C14	5.87/91.2	5.88/90.1	5.62/93.3	5.76/91.0
H15/C15	5.46/74.6	5.40/73.7	4.17/75.5|6.01 ^5^	4.28/74.6|5.69 ^5^
H16/C16	5.12/74.9	5.08/74.8	3.63/75.5|4.61 ^5^	3.74/75.5|4.87 ^5^
H17/C17	4.19/78.9	4.11/78.7	3.99/80.2	3.92/80.5
H18/C18	1.43/18.2	1.39/18.4	1.42/18.2	1.36/18.7

^1^ Due to the overlapping signals, one cannot assign them to the C2/C4 form **A** or C4 form **B**; ^2^ In square brackets are the ^1^*J*(C5-F) and ^2^*J*(C4-F)/^2^*J*(C6-F) spin-spin coupling constants; ^3^ Not determined due to the signal overlap; ^4^ Not observed in the ^1^H-^15^N g-HMBC experiment; value predicted from the linear regression in parentheses and in italic, details in Tables S5 and S7; ^5^ The ^1^H-NMR chemical shifts of hydroxyl protons at C15/C16.

**Table 3:**
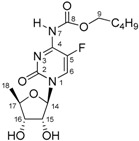
The ^1^H/^13^C/^15^N chemical shifts of capecitabine in H_2_O/D_2_O (298 K) and capecitabine acidified with HClO_4_ in THF-*d*_8_ (283 K).

	D_2_O	THF + HClO_4_
N1	−225.0	−221.0
C2	157.0	145.6
N3	(−*158.4*) ^1^	−225.8
C4	157.1 [12.6 Hz] ^2^	152.9 [20.2 Hz] ^2^
C5	140.5 [245.2 Hz] ^2^	136.4 [233.4 Hz] ^2^
F	−163.2	−165.8
H6/C6	8.06/131.3 [33.4 Hz] ^2^	8.36 [6.0 Hz]/135.4 [34.6 Hz] ^2^
N7	(−*271.6*) ^1^	−259.4
C8	155.6	154.3
H9/C9	4.23/70.0	4.32/69.2
H10/C10	1.70/30.3	1.73/28.9
H11/C11	1.35/30.0	1.38/28.6
H12/C12	1.34/24.4	1.36/23.1
H13/C13	0.88/16.0	0.90/14.3
H14/C14	5.78/94.6	5.69/94.5
H15/C15	4.30/77.4	4.26/75.5
H16/C16	3.88/76.9	3.81/75.1
H17/C17	4.21/82.5	4.05/81.3
H18/C18	1.47/20.1	1.42/18.1

^1^ Unrecorded in the ^1^H-^15^N g-HMBC experiment; in parentheses and in italics the predicted values from the linear regression; details in Tables S5 and S8; ^2^ In square brackets: the ^1^*J*(C5-F) and ^2^*J*(C4-F)/^2^*J*(C6-F) spin-spin coupling constants.

## Supplementary Materials

The Supplementary Materials are available online.
