# Supplementary material for: Multinuclear NMR Measurements and DFT Calculations for Capecitabine Tautomeric Form Assignment in a Solution
Source: Molecules. 2018 Jan 13;23(1):161. doi: 10.3390/molecules23010161 (PMC6016955; doi:10.3390/molecules23010161)
Supplement: Supplementary file 1 [file molecules-23-00161-s001.zip › SupplementaryMaterials.docx]

SUPPLEMENTARY MATERIALS

**Table S1.** The DFT, B3LYP/6–311G(2d,2p) energies of the tautomeric forms in vacuum^1^.

| No. | amino **I** | imino **II** | **III** | **IV** |
| --- | --- | --- | --- | --- |
| 1 | –1300.702789 | –1300.707942 | –1300.667305 | –1300.666911 |
| 2 | 0.381410 | 0.381670 | 0.380889 | 0.380456 |
| 3 | –1300.321379 | –1300.326272 | –1300.286416 | –1300.286455 |
| 4 | –1300.296424 | –1300.301575 | –1300.261074 | –1300.261406 |
| 5 | –1300.295480 | –1300.300631 | –1300.260130 | –1300.260462 |
| 6 | –1300.379895 | –1300.384073 | –1300.345540 | –1300.344256 |
| 7 | 13.5 | 0.00 | 106.7 | 107.7 |
| 8 | –0.67 | 0.00 | –2.05 | –3.18 |
| 9 | 12.8 | 0.00 | 104.6 | 104.6 |
| 10 | 13.5 | 0.00 | 106.3 | 105.5 |
| 11 | 13.5 | 0.00 | 106.3 | 105.5 |
| 12 | 11.0 | 0.00 | 101.2 | 104.6 |

^1^ Rows No. 1–6 in Hartree (1 Hartree = 2625.5 kJ/mol), 7–12 in kJ/mol.

No. 1: the electronic energy

2: the ZPE energy

3: the electronic + ZPE energy

4: the electronic + thermal energy

5: the electronic + thermal enthalpy

6: the electronic + free energy.

The 7–12 entries correspond to the energy differences (of 1–6) relative to the energy of the imino (**II**) form taken as the reference. The thermal contributions are calculated at T = 298.15 K.

**Table S2.** Relative electronic energies of the isolated amino (**I**) and imino (**II**) tautomeric forms
in kJ/mol predicted with various theoretical methods.

| Method | Energy difference (**I** – **II**) |
| --- | --- |
| B3LYP/6–31G(d,p) | 14.2 |
| B3LYP/6–311G(2d,2p) | 13.5 |
| B3LYP/6–311++G(2d,2p) | 12.9 |
| B3LYP/pcJ–1 | 11.1 |
| B3LYP/aug–cc–pVDZ | 14.0^1^ |
| wB97XD/pcJ–1 | 14.3 |
| RHF/6–311G(2d,2p) | 14.6 |

^1^ After the revision of the geometry optimization of Malinska *et al*. (**2014**) (the former result was 78.6 kJ/mol [6]).

**Table S3.** ^1^H isotropic shieldings (σ_ppm_) and chemical shifts (δ_ppm_) of H3/H7 and H6 hydrogen atoms predicted with the DFT B3LYP/6–311G(2d,2p) method.

| System | σ^1^ | δ^1^ |
| --- | --- | --- |
| isolated |  |  |
| amino(**I**) H7 | 24.95 | 6.86 |
| amino(**I**) H6 | 23.79 | 8.02 |
| imino(**II**) H3 | 19.28 | 12.53 |
| imino(**II**) H6 | 24.60 | 7.21 |
| binary complex^2^ |  |  |
| amino(**I**–DMSO) H7 | 21.24 | 10.57 |
| amino(**I**–DMSO) H6 | 23.56 | 8.25 |
| imino(**II**–DMSO) H3 | 19.49 | 12.32 |
| imino(**II**–DMSO) H6 | 23.90 | 7.91 |

^1^ The isotropic ^1^H shielding of TMS predicted with the B3LYP/6–311G(2d,2p) is 31.81 ppm.

^2^ The binary complexes used in the present work are visualized in Figures S7_a and S7_b.

**Table S4.** The effect of the solvent on the relative energy of **I** and **II** capecitabine tautomers predicted with the DFT method with the continuous solvent model, in kJ/mol.

| System | Energy difference (**I** – **II**) | |
| --- | --- | --- |
|  | wB97XD/pcJ–1 | B3LYP/6–311++G(2d,2p) |
| isolated | 14.3 | 12.9 |
| Encapsulated by THF | 2.47 | 2.26 |
| Encapsulated by DMSO | –0.63 | –0.75 |
| Encapsulated by H2O | –5.94 | –5.15 |
| (Me)^1^ Encapsulated by THF | 0.59 | — |

^1^ Energy difference (**2**–**3**) of capecitabine methyl derivatives.

**Table S5.** Linear regression parameters for the prediction of experimental NMR shieldings and coupling constants by the DFT (wB97XD/pcJ–1) method ^1,2^).

| Chemical shifts | | | | |
| --- | --- | --- | --- | --- |
| Nucleus | *a* (s.d.) | *R*^2^ | *n* | Pearson correlation |
| ^1^H (C6) | 0.904 (0.005) | 0.9998 | 6 | 0.958 |
| ^13^C (C5) | 0.947 (0.005) | 0.9998 | 7 | 0.786 |
| ^15^N (N1) | 0.998 (0.009) | 0.9996 | 6 | 0.942 |
| ^15^N (N3) | 0.946 (0.023) | 0.9977 | 5 | 0.983 |
| ^15^N (N7) | 0.930 (0.013) | 0.9994 | 4 | 0.998 |
| ^19^F | 0.908 (0.009) | 0.9995 | 6 | 0.669 |
| ^1^*J* (C5–F) coupling constants | | | | |
|  | 0.904 (0.006) | 0.9997 | 7 | 0.924 |

^1^ Linear regression omitting the intersection term is y = a·x, where y are the experimental shieldings/coupling constants, x are the corresponding DFT (wB97XD/pcJ–1) values, respectively, presented in Tables 1–3, and a is the slope, *R*^2^ is the determination coefficient and *n* is the number of data points included in the regression.

^2^ Inserting the DFT value (x) into the y = a·x equation made it possible to estimate (predict) the corresponding (lacking) experimental data. Such predicted values are given in Tables 1–3 in parentheses, in italics.

**Table S6.** ^19^F shielding (σ) and chemical shift (δ) predicted with the DFT at various levels of calculations, in ppm. Experimental ^19^F NMR data are included for comparison.

| **Tautomer** | **Method** | **σ** | **δ** | **Δ = δ(I) – δ(II)** |
| --- | --- | --- | --- | --- |
| **I** | B3LYP/6–31G(d,p) | 364.1 | –184.6 | –19.0 |
| **II** | B3LYP/6–31G(d,p) | 345.0 | –165.6 |  |
| **I** | B3LYP/6–311++G(2d,2p) | 351.6 | –194.9 | –4.0 |
| **II** | B3LYP/6–311++G(2d,2p) | 347.6 | –190.9 |  |
| **I** | wB97XD/pcJ–1 (DMSO) | 369.8 | –184.4 | –6.5 |
| **II** | wB97XD/pcJ–1 (DMSO) | 363.3 | –177.8 |  |
| **I** | wB97XD/pcJ–1 (THF) | 370.2 | –184.8 | –9.4 |
| **II** | wB97XD/pcJ–1 (THF) | 360.8 | –175.4 |  |
| **I** (N7–methyl) | wB97XD/pcJ–1 (THF) | 355.6 | –170.2 | +2.2 |
| **II** (N3–methyl) | wB97XD/pcJ–1 (THF) | 357.8 | –172.4 |  |
| **I** (F–5–cytosine) | wB97XD/pcJ–1 | 374.4 | –189.0 | –10.9 |
| **II** (F–5–cytosine) | wB97XD/pcJ–1 | 363.5 | –178.1 |  |
| **I** NMR (DMSO–d_6_) 298 K | experimental ^1^ |  | –159.8 | +3.5 |
| **II** NMR (DMSO–d_6_) 298 K | experimental ^1^ |  | –163.3 |  |
| **I** NMR (THF–d_8_) 298 K | experimental ^1^ |  | –162.6 | +2.1 |
| **II** NMR (THF–d_8_) 298 K | experimental ^1^ |  | –164.7 |  |
| **I** (N7–methyl) | experimental ^1^ |  | –154.5 | +6.5 |
| **II** (N3–methyl) | experimental ^1^ |  | –161.0 |  |

^1^ Present work.

**Table S7.** A comparison of the NMR and DFT chemical shifts in ppm and selected spin–spin coupling constants in Hz in square brackets (^1^*J*(C5–F) and ^2^*J*(C4–F)/^2^*J*(C6–F) ) for **I** and **II** tautomers of capecitabine and their methyl-derivatives (**2** and **3**).

|  | **2** | | **3** | | **A=I** | | **B=II** | |
| --- | --- | --- | --- | --- | --- | --- | --- | --- |
| **Atom** | **NMR** | **DFT** | **NMR** | **DFT** | **NMR** | **DFT** | **NMR** | **DFT** |
| N1 | –226.2 | –218.4 | –255.9 | –255.7 | –221.7 | –229.1 | –244.6 | –248.1 |
| C2 | 153 | 162.7 | 149.6 | 159.6 | 154.0^1^ | 163 | 147.4 | 157 |
| N3 | –123.9 | –124.5 | –245.6 | –246.3 | –139.3 | –159.6 | –236.5 | –246.7 |
| C4 | 159.2 | 169.7 | 145.6 | 152.1 | 154.4 | 162.4 | 154.0^1^ | 163.1 |
|  | [12.1] | [7.71] | [26.0] | [18.3] | [11.8] | [7.72] | [(25.9)] | [17.6] |
| C5 | 140.5 | 150.4 | 139.3 | 149.8 | 137.9 | 145 | 140.3 | 149.2 |
|  | [243.9] | [288.1] | [229.6] | [258.9] | [243.6] | [266.5] | [232.4] | [261.0] |
| C6 | 130.3 | 142.3 | 121.3 | 130.2 | 129.7 | 138.6 | 126.5 | 135.8 |
|  | [36.2] | [32.9] | [36.0] | [36.3] | [34.0] | [31.8] | [34.5] | [34.9] |
| N7 | –276.2 | –292 | –151.7 | –173.6 | –268.9 | –284.8 | (–198.4)^2^ | –206.4 |
| F | –154.5 | –170.2 | –161 | –172.4 | –161.5 | –184.8 | –163.3 | –175.4 |
| C8 | 154.5 | 161.4 | 160.4 | 167.5 | 151.7 | 156.8 | 164.7 | 173.2 |
| C9 | 67.8 | 71.2 | 66.4 | 70.7 | 66.5 | 71.4 | 66.3 | 71.5 |
| C10 | 29.2 | 30.5 | 29.4 | 30.6 | 29.4 | 30 | 29.4 | 30.1 |
| C11 | 28.8 | 28.6 | 29 | 28.6 | 28.9 | 30.2 | 29.2 | 30.5 |
| C12 | 23.2 | 25.1 | 23.2 | 25.4 | 23.5 | 25.1 | 23.6 | 25 |
| C13 | 14.3 | 14.1 | 14.3 | 14.1 | 14.7 | 14 | 14.7 | 14 |
| C14 | 91.2 | 100.9 | 90.1 | 100.7 | 93.3 | 100.7 | 91 | 100.8 |
| C15 | 74.6 | 84.6 | 73.7 | 84.9 | 75.5 | 84.5 | 74.7 | 84.8 |
| C16 | 74.9 | 81.6 | 74.8 | 81.7 | 75.5 | 81.7 | 75.5 | 81.7 |
| C17 | 78.9 | 90.8 | 78.7 | 91 | 80.2 | 90.5 | 80.5 | 91.2 |
| C18 | 18.2 | 20.7 | 18.4 | 20.4 | 18.2 | 20.6 | 18.7 | 20.3 |
| CH3 | 34.7 | 38.7 | 30.4 | 33.7 | – | – | – | – |
| H6 | 7.91 | 8.82 | 7.52 | 8.32 | 7.96 | 8.67 | 7.86 | 8.56 |
| H7 | – | – | – | – | 10.08 | 7.37 | 11.88 | 12.24 |
| H9 | 4.17 | 4.31 | 4.05 | 4.21 | 4.11 | 4.25 | 4.05 | 4.19 |
| H10 | 1.65 | 1.65 | 1.63 | 1.69 | 1.65 | 1.65 | 1.65 | 1.68 |
| H11 | 1.35 | 1.22 | 1.35 | 1.22 | 1.35 | 1.47 | 1.35 | 1.53 |
| H12 | 1.35 | 1.44 | 1.35 | 1.46 | 1.35 | 1.45 | 1.35 | 1.47 |
| H13 | 0.91 | 1.03 | 0.9 | 1.04 | 0.91 | 1.05 | 0.91 | 1.05 |
| H14 | 5.87 | 5.69 | 5.88 | 5.57 | 5.62 | 5.6 | 5.76 | 5.54 |
| H15 | 5.46 | 4.25 | 5.4 | 4.29 | 4.17 | 4.25 | 4.28 | 4.32 |
| H(O15) | – | 5.64 | – | 4.82 | 6.01 | 5.69 | 5.69 | 4.8 |
| H16 | 5.12 | 4.24 | 5.08 | 4.23 | 3.63 | 4.22 | 3.74 | 4.26 |
| H(O16) | – | 3.16 | – | 3.09 | 4.61 | 3.16 | 4.87 | 3.01 |
| H17 | 4.19 | 4.72 | 4.11 | 4.69 | 3.99 | 4.69 | 3.92 | 4.72 |
| H18 | 1.43 | 1.32 | 1.39 | 1.34 | 1.42 | 1.3 | 1.36 | 1.33 |
| H (CH_3_) | 3.29 | 3.29 | 3.27 | 3.36 | – | – | – | – |

^1^ Overlapping signals.

^2^ Linearly extrapolated (not determined experimentally due to overlapping signals).

**Table S8.** A comparison of the NMR and DFT chemical shifts in ppm and selected spin–spin coupling constants in Hz in square brackets (^1^*J*(C5–F) and ^2^*J*(C4–F)/^2^J(C6–F)) for tautomer **I** of capecitabine in H_2_O and HClO_4_. For comparison, DFT results for hypothetical (undetected in NMR measurements) tautomer **II** in H_2_O are given.

|  | **I in H_2_O** | | **II in H_2_O** | | **I in HClO_4_** | |
| --- | --- | --- | --- | --- | --- | --- |
| **Atom** | **NMR** | **DFT** | **NMR** | **DFT** | **NMR** | **DFT** |
| N1 | –225 | –226.7 | – | –243.9 | –221 | –218.4 |
| C2 | 157 | 164.9 | – | 158.1 | 145.6 | 153.2 |
| N3 | (–159.0)^1^ | –165.4 | – | –244.3 | –225.8 | –250.3 |
| C4 | 157.1 | 163.2 | – | 164.3 | 152.9 | 159.9 |
|  | [12.6] | [8.0] | – | [16.6] | [23.8] | [16.2] |
| C5 | 140.5 | 145.6 | – | 149 | 136.4 | 141.1 |
|  | [245.2] | [266.6] | – | [257.1] | [233.4] | [264.0] |
| C6 | 131.3 | 140.6 | – | 138.3 | 135.5 | 150.1 |
|  | [33.4] | [32.9] | – | [36.3] | [33.0] | [30.2] |
| N7 | (–272.3)^1^ | –283.3 | – | –214.5 | –259.4 | –282.4 |
| F | –163.2 | –182.2 | – | –177.1 | –165.8 | –181.9 |
| C8 | 155.6 | 160.6 | – | 174.4 | 154.3 | 160.7 |
| C9 | 70 | 72.9 | – | 72.2 | 69.2 | 77.7 |
| C10 | 30.3 | 29.5 | – | 29.7 | 28.9 | 29.6 |
| C11 | 30 | 30.2 | – | 30.5 | 28.6 | 30.3 |
| C12 | 24.4 | 24.8 | – | 24.9 | 23.1 | 24.8 |
| C13 | 16 | 13.8 | – | 13.8 | 14.3 | 13.8 |
| C14 | 94.6 | 101 | – | 101 | 94.5 | 104.1 |
| C15 | 77.4 | 84.5 | – | 84.8 | 75.5 | 84.1 |
| C16 | 76.9 | 82.4 | – | 82.2 | 75.1 | 81.9 |
| C17 | 82.5 | 91.1 | – | 91.6 | 81.3 | 92.6 |
| C18 | 20.1 | 20.2 | – | 19.8 | 18.1 | 20.1 |
| H6 | 8.06 | 8.89 | – | 8.78 | 8.36 | 9.43 |
| H7 | – | 7.79 | – | – | 10.08 | 8.09 |
| H9 | 4.23 | 4.36 | – | 4.27 | 4.32 | 4.64 |
| H10 | 1.7 | 1.77 | – | 1.72 | 1.73 | 1.82 |
| H11 | 1.35 | 1.48 | – | 1.51 | 1.38 | 1.51 |
| H12 | 1.34 | 1.45 | – | 1.47 | 1.36 | 1.49 |
| H13 | 0.88 | 1.05 | – | 1.06 | 0.9 | 1.08 |
| H14 | 5.78 | 5.81 | – | 5.75 | 5.69 | 5.76 |
| H15 | 4.3 | 4.49 | – | 4.54 | 4.26 | 4.45 |
| H(O15) | – | 5.59 | – | 4.83 | 6.01 | 4.17 |
| H16 | 3.88 | 4.39 | – | 4.4 | 3.81 | 4.4 |
| H(O16) | – | 3.48 | – | 3.44 | 4.61 | 2.95 |
| H17 | 4.21 | 4.77 | – | 4.77 | 4.05 | 4.88 |
| H18 | 1.47 | 1.41 | – | 1.39 | 1.42 | 1.34 |

^1^ Linearly extrapolated (not determined experimentally).

**Table S9.** Linear regression^1^ for ^15^N chemical shifts discussed in the present work.

| No**.** | **Molecule** | **Atom** | **NMR** | **DFT** |
| --- | --- | --- | --- | --- |
| 1 | **I** (THF) | N1 | –221.7 | –229.1 |
| 2 | **I** (THF) | N3 | –139.3 | –159.6 |
| 3 | **I** (THF) | N7 | –268.9 | –284.8 |
| 4 | **II** (THF) | N1 | –244.6 | –248.1 |
| 5 | **II** (THF) | N3 | –236.5 | –246.5 |
| 6 | **II** (THF) | N7 | — | –206.4 |
| 7 | **I** (H2O) | N1 | –225.0 | –226.7 |
| 8 | **I** (H2O) | N3 | — | –165.4 |
| 9 | **I** (H2O) | N7 | — | –283.3 |
| 10 | **I** (HClO4) | N1 | –221.0 | –218.4 |
| 11 | **I** (HClO4) | N3 | –225.8 | –250.3 |
| 12 | **I** (HClO4) | N7 | –259.4 | –282.4 |
| 13 | **2** (THF) | N1 | –226.2 | –218.4 |
| 14 | **2** (THF) | N3 | –123.9 | –124.5 |
| 15 | **2** (THF) | N7 | –276.2 | –292.0 |
| 16 | **3** (THF) | N1 | –255.9 | –255.8 |
| 17 | **3** (THF) | N3 | –245.6 | –255.8 |
| 18 | **3** (THF) | N7 | –151.7 | –173.6 |

^1^ The linear regression of NMR (y) vs. DFT (x): y = a·x, where a = 0.96112 +/– 0.01143,
95% C.I. = (0.93660, 0.98564), *R*^2^ = 0.9980, see also Figure S9.

**Table S10.** Input data for linear regression of Table S5: Experimental NMR and theoretical DFT ^1^H chemical shifts, in ppm, discussed in the present work. The selected hydrogens are located in the central region of the capecitabine molecule.

| **No.** | **Molecule** | **Atom** | **NMR** | **DFT** **^3^** |
| --- | --- | --- | --- | --- |
| 1 | **I** (THF) | H7 ^1^ | 10.08 | 7.37 (10.21) |
| 2 | **I** (THF) | H6 | 7.96 | 8.67 (8.15) |
| 3 | **I** (THF) | H14 | 5.62 | 5.60 (5.86) |
| 4 | **II** (THF) | H3 ^1^ | 11.88 | 12.24 (11.85) |
| 5 | **II** (THF) | H6 | 7.86 | 8.56 (7.47) |
| 6 | **II** (THF) | H14 | 5.76 | 5.54 (5.01) |
| 7 | **I** (H_2_O) | H7 ^1^ | n.a.^2^ | 7.79 (10.46) |
| 8 | **I** (H_2_O) | H6 | 8.06 | 8.89 (8.33) |
| 9 | **I** (H_2_O) | H14 | 5.78 | 5.81 (5.92) |
| 10 | **I** (HClO_4_+THF) | H7/H3 ^1^ | n.a.^2^ | 8.09/12.02 ^4^ |
| 11 | **I** (HClO_4_+THF) | H6 | 8.36 | 9.43 |
| 12 | **I** (HClO_4_+THF) | H14 | 5.69 | 5.76 |
| 13 | **2** (THF) | H6 | 7.91 | 8.82 |
| 14 | **2** (THF) | H14 | 5.87 | 5.69 |
| 15 | **3** (THF) | H6 | 7.52 | 8.32 |
| 16 | **3** (THF) | H14 | 5.88 | 5.57 |

^1^ Hydrogens involved in the intermolecular hydrogen bonds.

^2^ Not available.

^3^ The DFT wB97XD/pcJ–1 with the SMD model of the solvent; in parentheses the binary capecitabine–THF and ternary capecitabine–(H_2_O)_2_ complexes from the DFT B3LYP/6–311G(2d,2p) calculations.

^4^ 8.07 ppm (H7); 12.02 ppm (H3).

**Table S11.** Input data for linear regression of Table S5: Experimental NMR and theoretical DFT ^13^C chemical shifts discussed in the present work. The selected carbons are located nearby the central region of the capecitabine molecule.

| **No.** | **Molecule** | **Atom** | **NMR** | **DFT** **^1^** |
| --- | --- | --- | --- | --- |
| 1 | **I** (THF) | C2 | 154.0 | 163.0 (160.6) |
| 2 | **I** (THF) | C4 | 154.4 | 162.4 (157.4) |
| 3 | **I** (THF) | C5 | 137.9 | 145.0 (144.8) |
| 4 | **I** (THF) | C6 | 129.7 | 138.6 (133.5) |
| 5 | **I** (THF) | C8 | 151.7 | 156.8 (156.1) |
| 6 | **II** (THF) | C2 | 147.4 | 157.0 (152.6) |
| 7 | **II** (THF) | C4 | 154.0 | 163.1 (158.1) |
| 8 | **II** (THF) | C5 | 140.3 | 149.2 (148.0) |
| 9 | **II** (THF) | C6 | 126.5 | 135.8 (136.9) |
| 10 | **II** (THF) | C8 | 164.7 | 173.2 (170.6) |
| 11 | **I** (H_2_O) | C2 | 157.0 | 164.9 (160.7) |
| 12 | **I** (H_2_O) | C4 | 157.1 | 163.2 (158.5) |
| 13 | **I** (H_2_O) | C5 | 140.5 | 145.6 (145.4) |
| 14 | **I** (H_2_O) | C6 | 131.3 | 140.6 (135.2) |
| 15 | **I** (H_2_O) | C8 | 155.6 | 160.6 (158.3) |
| 16 | **I** (HClO_4_+THF) | C2 | 145.6 | 153.2 |
| 17 | **I** (HClO_4_+THF) | C4 | 152.9 | 159.9 |
| 18 | **I** (HClO_4_+THF) | C5 | 136.4 | 141.1 |
| 19 | **I** (HClO_4_+THF) | C6 | 135.5 | 150.1 |
| 20 | **I** (HClO_4_+THF) | C8 | 154.3 | 160.7 |
| 21 | **2** (THF) | C2 | 153.0 | 162.7 |
| 22 | **2** (THF) | C4 | 159.2 | 169.7 |
| 23 | **2** (THF) | C5 | 140.5 | 150.4 |
| 24 | **2** (THF) | C6 | 130.3 | 142.3 |
| 25 | **2** (THF) | C8 | 154.5 | 161.4 |
| 26 | **3** (THF) | C2 | 149.6 | 159.6 |
| 27 | **3** (THF) | C4 | 145.6 | 152.1 |
| 28 | **3** (THF) | C5 | 139.3 | 149.8 |
| 29 | **3** (THF) | C6 | 121.3 | 130.2 |
| 30 | **3** (THF) | C8 | 160.4 | 167.5 |

^1^ the DFT wB97XD/pcJ–1 with the SMD model of the solvent; in parentheses the binary capecitabine-THF and ternary capecitabine–(H_2_O)_2_ complexes from the DFT B3LYP/6–311G(2d,2p) calculations. The local hydrogen bond interactions modelled in complexes bring the theoretical chemical shifts closer to the experimental NMR values.

**Figure S1.** The ^15^N NMR chemical shifts of cytidine **4** and 5’–deoxy–5–fluorocytidine **5** in DMSO-d_6_ [present work].


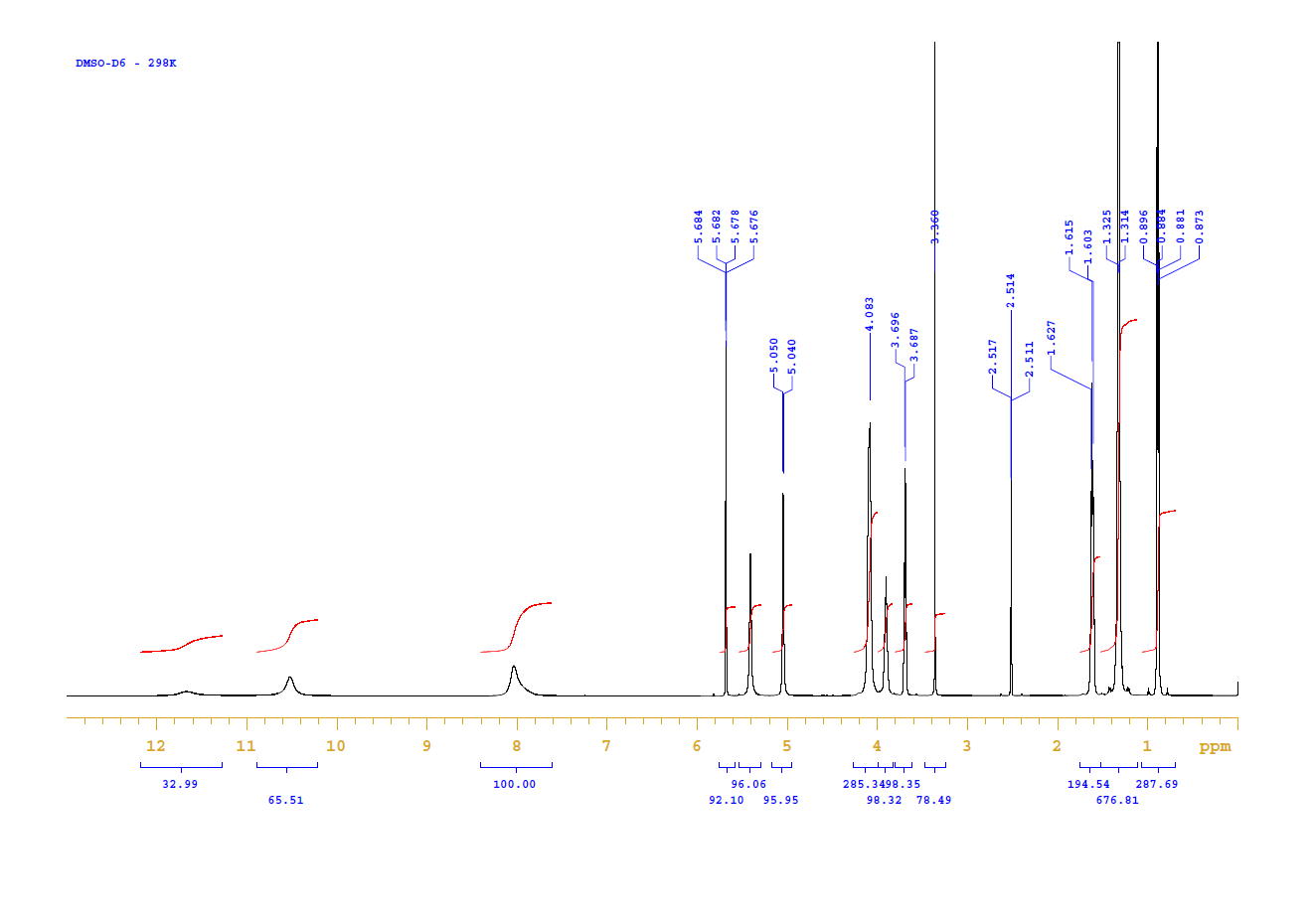


**Figure S2.** The ^1^H NMR spectrum of capecitabine in DMSO–d_6_ at 298 K. Tentatively, the signal at 10.52 ppm (integral 66) is assigned to form **A**, while 11.68 ppm (integral 33) to form **B**.

**Figure S3.** The ^19^F NMR spectrum of capecitabine in DMSO–d_6_ at 298 K.

**Figure S4.** The ^13^C NMR spectrum of capecitabine (DMSO–d_6_ solution, 298 K, overnight accumulation).

**Figure S5.** The ^1^H NMR temperature spectra (containing integrals) of the capecitabine **1** in THF–d_8_
(only the down–field region is presented).

**Figure S6.** The ^19^F NMR temperature spectra (containing integrals) of capecitabine **1** in THF–d_8_.


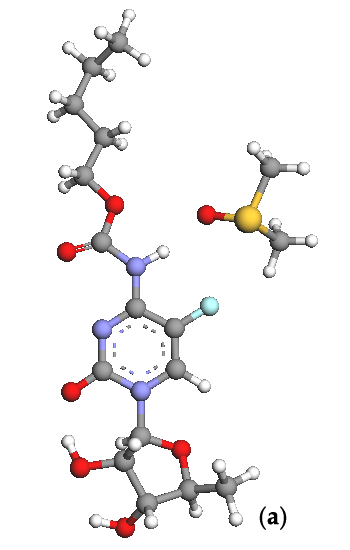

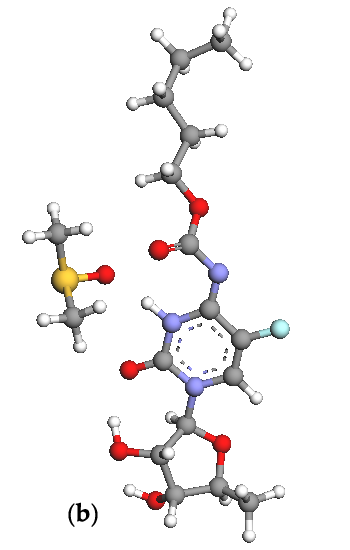


**Figure S7.** Binary complex: (**a**) capecitabine (**I)**–DMSO; (**b**) capecitabine (**II)**–DMSO.


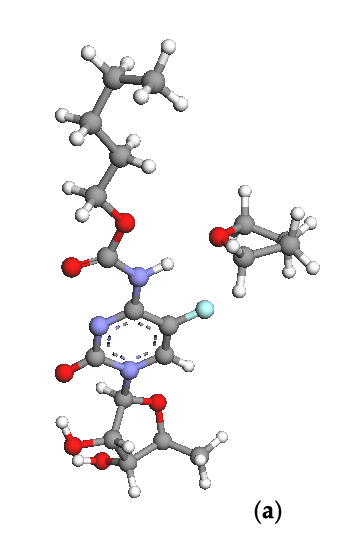

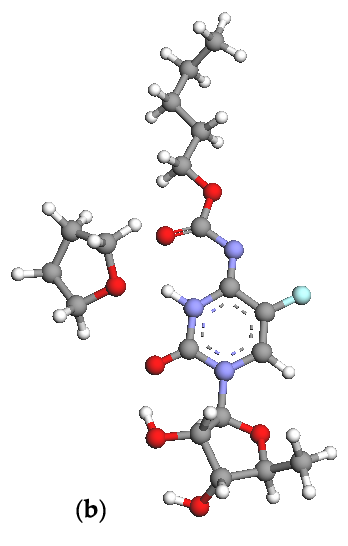


**Fig. S8.** Binary complex: (**a**) capecitabine (**I)**–THF; (**b**) capecitabine (**II)**–THF.


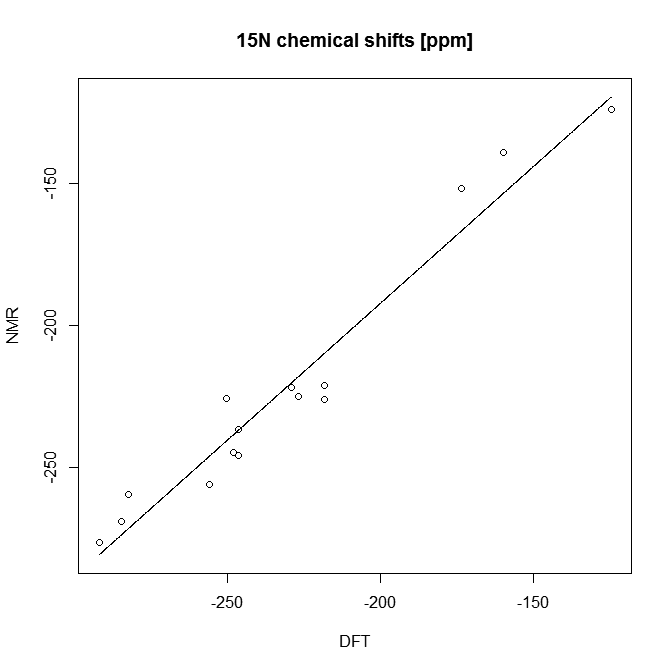


**Fig. S9.** The linear regression of ^15^N NMR chemical shifts: experimental (*y*) vs. DFT (*x*): *y* = 0.96112·*x*.
