# Supplementary material for: Multinuclear NMR Measurements and DFT Calculations for Capecitabine Tautomeric Form Assignment in a Solution
Source: Molecules. 2018 Jan 13;23(1):161. doi: 10.3390/molecules23010161 (PMC6016955; doi:10.3390/molecules23010161)
Supplement: Supplementary file 1 [file molecules-23-00161-s001.zip › TableS1.docx]

**Table S1.** The DFT, B3LYP/6–311G(2d,2p) energies of the tautomeric forms in vacuum^1^.

| No. | amino **I** | imino **II** | **III** | **IV** |
| --- | --- | --- | --- | --- |
| 1 | –1300.702789 | –1300.707942 | –1300.667305 | –1300.666911 |
| 2 | 0.381410 | 0.381670 | 0.380889 | 0.380456 |
| 3 | –1300.321379 | –1300.326272 | –1300.286416 | –1300.286455 |
| 4 | –1300.296424 | –1300.301575 | –1300.261074 | –1300.261406 |
| 5 | –1300.295480 | –1300.300631 | –1300.260130 | –1300.260462 |
| 6 | –1300.379895 | –1300.384073 | –1300.345540 | –1300.344256 |
| 7 | 13.5 | 0.00 | 106.7 | 107.7 |
| 8 | –0.67 | 0.00 | –2.05 | –3.18 |
| 9 | 12.8 | 0.00 | 104.6 | 104.6 |
| 10 | 13.5 | 0.00 | 106.3 | 105.5 |
| 11 | 13.5 | 0.00 | 106.3 | 105.5 |
| 12 | 11.0 | 0.00 | 101.2 | 104.6 |

^1^ Rows No. 1–6 in Hartree (1 Hartree = 2625.5 kJ/mol), 7–12 in kJ/mol.

No. 1: the electronic energy

2: the ZPE energy

3: the electronic + ZPE energy

4: the electronic + thermal energy

5: the electronic + thermal enthalpy

6: the electronic + free energy.

The 7–12 entries correspond to the energy differences (of 1–6) relative to the energy of the imino (**II**) form taken as the reference. The thermal contributions are calculated at T = 298.15 K.
