# Supplementary material for: Multinuclear NMR Measurements and DFT Calculations for Capecitabine Tautomeric Form Assignment in a Solution
Source: Molecules. 2018 Jan 13;23(1):161. doi: 10.3390/molecules23010161 (PMC6016955; doi:10.3390/molecules23010161)
Supplement: Supplementary file 1 [file molecules-23-00161-s001.zip › TableS10.docx]

**Table S10.** Input data for linear regression of Table S5: Experimental NMR and theoretical DFT ^1^H chemical shifts, in ppm, discussed in the present work. The selected hydrogens are located in the central region of the capecitabine molecule.

| **No.** | **Molecule** | **Atom** | **NMR** | **DFT ^3^** |
| --- | --- | --- | --- | --- |
| 1 | **I** (THF) | H7 ^1^ | 10.08 | 7.37 (10.21) |
| 2 | **I** (THF) | H6 | 7.96 | 8.67 (8.15) |
| 3 | **I** (THF) | H14 | 5.62 | 5.60 (5.86) |
| 4 | **II** (THF) | H3 ^1^ | 11.88 | 12.24 (11.85) |
| 5 | **II** (THF) | H6 | 7.86 | 8.56 (7.47) |
| 6 | **II** (THF) | H14 | 5.76 | 5.54 (5.01) |
| 7 | **I** (H_2_O) | H7 ^1^ | n.a.^2^ | 7.79 (10.46) |
| 8 | **I** (H_2_O) | H6 | 8.06 | 8.89 (8.33) |
| 9 | **I** (H_2_O) | H14 | 5.78 | 5.81 (5.92) |
| 10 | **I** (HClO_4_+THF) | H7/H3 ^1^ | n.a.^2^ | 8.09/12.02 ^4^ |
| 11 | **I** (HClO_4_+THF) | H6 | 8.36 | 9.43 |
| 12 | **I** (HClO_4_+THF) | H14 | 5.69 | 5.76 |
| 13 | **2** (THF) | H6 | 7.91 | 8.82 |
| 14 | **2** (THF) | H14 | 5.87 | 5.69 |
| 15 | **3** (THF) | H6 | 7.52 | 8.32 |
| 16 | **3** (THF) | H14 | 5.88 | 5.57 |

^1^ Hydrogens involved in the intermolecular hydrogen bonds.

^2^ Not available.

^3^ The DFT wB97XD/pcJ–1 with the SMD model of the solvent; in parentheses the binary capecitabine–THF and ternary capecitabine–(H_2_O)_2_ complexes from the DFT B3LYP/6–311G(2d,2p) calculations.

^4^ 8.07 ppm (H7); 12.02 ppm (H3).
