# Supplementary material for: Multinuclear NMR Measurements and DFT Calculations for Capecitabine Tautomeric Form Assignment in a Solution
Source: Molecules. 2018 Jan 13;23(1):161. doi: 10.3390/molecules23010161 (PMC6016955; doi:10.3390/molecules23010161)
Supplement: Supplementary file 1 [file molecules-23-00161-s001.zip › TableS11.docx]

**Table S11.** Input data for linear regression of Table S5: Experimental NMR and theoretical DFT ^13^C chemical shifts discussed in the present work. The selected carbons are located nearby the central region of the capecitabine molecule.

| **No.** | **Molecule** | **Atom** | **NMR** | **DFT ^1^** |
| --- | --- | --- | --- | --- |
| 1 | **I** (THF) | C2 | 154.0 | 163.0 (160.6) |
| 2 | **I** (THF) | C4 | 154.4 | 162.4 (157.4) |
| 3 | **I** (THF) | C5 | 137.9 | 145.0 (144.8) |
| 4 | **I** (THF) | C6 | 129.7 | 138.6 (133.5) |
| 5 | **I** (THF) | C8 | 151.7 | 156.8 (156.1) |
| 6 | **II** (THF) | C2 | 147.4 | 157.0 (152.6) |
| 7 | **II** (THF) | C4 | 154.0 | 163.1 (158.1) |
| 8 | **II** (THF) | C5 | 140.3 | 149.2 (148.0) |
| 9 | **II** (THF) | C6 | 126.5 | 135.8 (136.9) |
| 10 | **II** (THF) | C8 | 164.7 | 173.2 (170.6) |
| 11 | **I** (H_2_O) | C2 | 157.0 | 164.9 (160.7) |
| 12 | **I** (H_2_O) | C4 | 157.1 | 163.2 (158.5) |
| 13 | **I** (H_2_O) | C5 | 140.5 | 145.6 (145.4) |
| 14 | **I** (H_2_O) | C6 | 131.3 | 140.6 (135.2) |
| 15 | **I** (H_2_O) | C8 | 155.6 | 160.6 (158.3) |
| 16 | **I** (HClO_4_+THF) | C2 | 145.6 | 153.2 |
| 17 | **I** (HClO_4_+THF) | C4 | 152.9 | 159.9 |
| 18 | **I** (HClO_4_+THF) | C5 | 136.4 | 141.1 |
| 19 | **I** (HClO_4_+THF) | C6 | 135.5 | 150.1 |
| 20 | **I** (HClO_4_+THF) | C8 | 154.3 | 160.7 |
| 21 | **2** (THF) | C2 | 153.0 | 162.7 |
| 22 | **2** (THF) | C4 | 159.2 | 169.7 |
| 23 | **2** (THF) | C5 | 140.5 | 150.4 |
| 24 | **2** (THF) | C6 | 130.3 | 142.3 |
| 25 | **2** (THF) | C8 | 154.5 | 161.4 |
| 26 | **3** (THF) | C2 | 149.6 | 159.6 |
| 27 | **3** (THF) | C4 | 145.6 | 152.1 |
| 28 | **3** (THF) | C5 | 139.3 | 149.8 |
| 29 | **3** (THF) | C6 | 121.3 | 130.2 |
| 30 | **3** (THF) | C8 | 160.4 | 167.5 |

^1^ the DFT wB97XD/pcJ–1 with the SMD model of the solvent; in parentheses the binary capecitabine-THF and ternary capecitabine–(H_2_O)_2_ complexes from the DFT B3LYP/6–311G(2d,2p) calculations. The local hydrogen bond interactions modelled in complexes bring the theoretical chemical shifts closer to the experimental NMR values.
