# Supplementary material for: Multinuclear NMR Measurements and DFT Calculations for Capecitabine Tautomeric Form Assignment in a Solution
Source: Molecules. 2018 Jan 13;23(1):161. doi: 10.3390/molecules23010161 (PMC6016955; doi:10.3390/molecules23010161)
Supplement: Supplementary file 1 [file molecules-23-00161-s001.zip › TableS2.docx]

**Table S2.** Relative electronic energies of the isolated amino (**I**) and imino (**II**) tautomeric forms
in kJ/mol predicted with various theoretical methods.

| Method | Energy difference (**I** – **II**) |
| --- | --- |
| B3LYP/6–31G(d,p) | 14.2 |
| B3LYP/6–311G(2d,2p) | 13.5 |
| B3LYP/6–311++G(2d,2p) | 12.9 |
| B3LYP/pcJ–1 | 11.1 |
| B3LYP/aug–cc–pVDZ | 14.0 ^1^ |
| wB97XD/pcJ–1 | 14.3 |
| RHF/6–311G(2d,2p) | 14.6 |

^1^ After the revision of the geometry optimization of Malinska *et al*. (**2014**) (the former result was 78.6 kJ/mol [6]).
