# Supplementary material for: Multinuclear NMR Measurements and DFT Calculations for Capecitabine Tautomeric Form Assignment in a Solution
Source: Molecules. 2018 Jan 13;23(1):161. doi: 10.3390/molecules23010161 (PMC6016955; doi:10.3390/molecules23010161)
Supplement: Supplementary file 1 [file molecules-23-00161-s001.zip › TableS3.docx]

**Table S3.** ^1^H isotropic shieldings (σ, ppm) and chemical shifts (δ, ppm) of H3/H7 and H6 hydrogen atoms predicted with the DFT B3LYP/6–311G(2d,2p) method.

| **System** | **σ^1^** | **δ^1^** |
| --- | --- | --- |
| isolated |  |  |
| amino(**I**) H7 | 24.95 | 6.86 |
| amino(**I**) H6 | 23.79 | 8.02 |
| imino(**II**) H3 | 19.28 | 12.53 |
| imino(**II**) H6 | 24.60 | 7.21 |
| Binary/ternary complexes^2^ |  |  |
| amino(**I**–THF) H7 | 21.60 | 10.21 |
| amino(**I**–THF) H6 | 23.66 | 8.15 |
| imino(**II**–THF) H3 | 19.96 | 11.85 |
| imino(**II**–THF) H6 | 24.34 | 7.47 |
| amino(**I**–DMSO) H7 | 21.24 | 10.57 |
| amino(**I**–DMSO) H6 | 23.56 | 8.25 |
| imino(**II**–DMSO) H3 | 19.49 | 12.32 |
| imino(**II**–DMSO) H6 | 23.90 | 7.91 |
| amino(**I**–(H_2_O)_2_) H7 | 21.35 | 10.46 |
| amino(**I**–(H_2_O)_2_) H6 | 23.48 | 8.33 |

^1^ The isotropic ^1^H and ^13^C shieldings of TMS predicted with the B3LYP/6–311G(2d,2p) are 31.81 and 183.86 ppm, respectively.

^2^ The binary/ternary complexes used in the present work are visualized in Figures: S7a (**I**–THF) , S7b (**II**–THF), S7c (**I**–DMSO), S7d (**II**–DMSO), S7e (**I**–(H_2_O)_2_ ).
