# Supplementary material for: Multinuclear NMR Measurements and DFT Calculations for Capecitabine Tautomeric Form Assignment in a Solution
Source: Molecules. 2018 Jan 13;23(1):161. doi: 10.3390/molecules23010161 (PMC6016955; doi:10.3390/molecules23010161)
Supplement: Supplementary file 1 [file molecules-23-00161-s001.zip › TableS4.docx]

**Table S4.** The effect of the solvent on the relative energy of **I** and **II** capecitabine tautomers predicted with the DFT method with the continuous solvent model, in kJ/mol.

| **System** | **Energy difference (I – II)** | |
| --- | --- | --- |
|  | **wB97XD/pcJ–1** | **B3LYP/6–311++G(2d,2p)** |
| isolated | 14.3 | 12.9 |
| Encapsulated by THF | 2.47 | 2.26 |
| Encapsulated by DMSO | –0.63 | –0.75 |
| Encapsulated by H_2_O | –5.94 | –5.15 |
| (N7/N3-methyl-capecitabine)^1^  encapsulated by THF | 0.60 | — |

^1^ Energy difference (**2**–**3**) of capecitabine methyl derivatives.
