# Supplementary material for: Multinuclear NMR Measurements and DFT Calculations for Capecitabine Tautomeric Form Assignment in a Solution
Source: Molecules. 2018 Jan 13;23(1):161. doi: 10.3390/molecules23010161 (PMC6016955; doi:10.3390/molecules23010161)
Supplement: Supplementary file 1 [file molecules-23-00161-s001.zip › TableS5.docx]

**Table S5.** Linear regression parameters for the prediction of experimental NMR shieldings and coupling constants by the DFT (wB97XD/pcJ–1) method ^1,2,3^.

| **Chemical shifts** | | | | | | |
| --- | --- | --- | --- | --- | --- | --- |
| Nucleus | *a* [ppm] | *b* | *RMSE* [ppm] | *R*^2^ | (*PRESS*/*n*)^1/2^ | *n* |
| ^1^H | 1.8248 | 0.6967 | 0.11 | 0.99216 | 0.12 | 12 |
| ^13^C | –11.9671 | 1.0237 | 2.22 | 0.9625 | 2.27 | 30 |
| ^15^N | 0.2996 | 0.9597 | 10.47 | 0.95316 | 11.26 | 15 |
| ^1^*J* (C5–F) coupling constants | | | | | | |
| C5–F | –254.63 | 1.8665 | 2.82 | 0.8537 | 3.04 | 7 |

^1^ Linear regression *y = a + b·x,* where *y* are the experimental shieldings/coupling constants, *x* are the corresponding DFT (wB97XD/pcJ–1) values presented in Tables 1–3, and *a* is the intersect, *b* is the slope, RMSE is the root mean squares error, *R*^2^ is the determination coefficient and *n* is the number of data points included in the regression.

^2^ Inserting the DFT value (*x*) into the *y = a + b·x* equation it is possible to estimate (predict) the lacking experimental data. Such predicted values are given in Tables 1–3 in parentheses, in italics.

^3^ Only atoms present in the central region of the capecitabine molecule were included in the linear regression:

^1^H: Table S10: entry 2,3,5,6,8,9,11–16; entries 1,4 were omitted because the DFT method applied for calculations in Table S10 did not include hydrogen bond with THF,

^13^C: Table S11: entry 1–30; ^15^N: Table S9, entry 1–5,7,10–18; ^1^*J*(C5–F): Table 1–3, Tables S7, S8; *a*, *RMSE* in Hz;

The prediction power of the present linear regression can be characterized by the quantity:

(*PRESS/n*)^½^ = (*1/n Σ_i_ (y_i_- ŷ_i_)^2^ / (1-h_ii_)^2^* )^½^, in ppm, which is the square root of the mean predicted residual sum of squares, and where *ŷ_i_* is the *i*th predicted *y* based on linear regression, *h_ii_* is the *i*th diagonal element of the hat-matrix. Dividing this quantity by (absolute) mean of *y* one can estimate the percent deviation of the regression line from the experimental values: about 5 % for δ (^15^N), and less than 1.7 % for δ (^1^H), δ (^13^C) and ^1^*J* (C5–F).

A reasonably good correlation between the quantum mechanically predicted ^1^H, ^13^C chemical shifts and spin–spin coupling constants of carbon and fluorine has been also obtained in dozens of papers [32–34].
