# Supplementary material for: Multinuclear NMR Measurements and DFT Calculations for Capecitabine Tautomeric Form Assignment in a Solution
Source: Molecules. 2018 Jan 13;23(1):161. doi: 10.3390/molecules23010161 (PMC6016955; doi:10.3390/molecules23010161)
Supplement: Supplementary file 1 [file molecules-23-00161-s001.zip › TableS6.docx]

**Table S6.** ^19^F shielding (σ) and chemical shift (δ) predicted with the DFT at various levels of calculations, in ppm. Experimental ^19^F NMR data are included for comparison.

| **Tautomer** | **Method** | **σ** | **δ** | **Δ = δ(I) – δ(II)** |
| --- | --- | --- | --- | --- |
| **I** | B3LYP/6–31G(d,p) | 364.1 | –184.6 | –19.0 |
| **II** | B3LYP/6–31G(d,p) | 345.0 | –165.6 |  |
| **I** | B3LYP/6–311++G(2d,2p) | 351.6 | –194.9 | –4.0 |
| **II** | B3LYP/6–311++G(2d,2p) | 347.6 | –190.9 |  |
| **I** | wB97XD/pcJ–1 (DMSO) | 369.8 | –184.4 | –6.5 |
| **II** | wB97XD/pcJ–1 (DMSO) | 363.3 | –177.8 |  |
| **I** | wB97XD/pcJ–1 (THF) | 370.2 | –184.8 | –9.4 |
| **II** | wB97XD/pcJ–1 (THF) | 360.8 | –175.4 |  |
| **I** (N7–methyl) | wB97XD/pcJ–1 (THF) | 355.6 | –170.2 | +2.2 |
| **II** (N3–methyl) | wB97XD/pcJ–1 (THF) | 357.8 | –172.4 |  |
| **I** (F–5–cytosine) | wB97XD/pcJ–1 | 374.4 | –189.0 | –10.9 |
| **II** (F–5–cytosine) | wB97XD/pcJ–1 | 363.5 | –178.1 |  |
| **I** NMR (DMSO–d_6_) | experimental ^1^ |  | –159.8 | +3.5 |
| **II** NMR (DMSO–d_6_) | experimental ^1^ |  | –163.3 |  |
| **I** NMR (THF–d_8_) | experimental ^1^ |  | –162.6 | +2.1 |
| **II** NMR (THF–d_8_) | experimental ^1^ |  | –164.7 |  |
| **I** (N7–methyl, in THF–d_8_) | experimental ^1^ |  | –154.5 | +6.5 |
| **II** (N3–methyl, in THF–d_8_) | experimental ^1^ |  | –161.0 |  |

^1^ Present work.
