# Supplementary material for: Multinuclear NMR Measurements and DFT Calculations for Capecitabine Tautomeric Form Assignment in a Solution
Source: Molecules. 2018 Jan 13;23(1):161. doi: 10.3390/molecules23010161 (PMC6016955; doi:10.3390/molecules23010161)
Supplement: Supplementary file 1 [file molecules-23-00161-s001.zip › TableS7.docx]

**Table S7.** Expanded Table 2: A comparison of the NMR and DFT chemical shifts in ppm and selected spin–spin coupling constants in Hz in square brackets (^1^*J*(C5–F) and ^2^*J*(C4–F)/^2^*J*(C6–F) ) for **I** and **II** tautomers of capecitabine and their methyl-derivatives (**2** and **3**).

|  | **2** | | **3** | | **A=I** | | **B=II** | |
| --- | --- | --- | --- | --- | --- | --- | --- | --- |
| **Atom** | **NMR** | **DFT** | **NMR** | **DFT** | **NMR** | **DFT** | **NMR** | **DFT** |
| N1 | –226.2 | –218.4 | –255.9 | –255.7 | –221.7 | –229.1 | –244.6 | –248.1 |
| C2 | 153 | 162.7 | 149.6 | 159.6 | 154.0^1^ | 163 | 147.4 | 157 |
| N3 | –123.9 | –124.5 | –245.6 | –246.3 | –139.3 | –159.6 | –236.5 | –246.7 |
| C4 | 159.2 | 169.7 | 145.6 | 152.1 | 154.4 | 162.4 | 154.0^1^ | 163.1 |
|  | [12.1] | [7.71] | [26.0] | [18.3] | [11.8] | [7.72] | [(25.9)] | [17.6] |
| C5 | 140.5 | 150.4 | 139.3 | 149.8 | 137.9 | 145 | 140.3 | 149.2 |
|  | [243.9] | [288.1] | [229.6] | [258.9] | [243.6] | [266.5] | [232.4] | [261.0] |
| C6 | 130.3 | 142.3 | 121.3 | 130.2 | 129.7 | 138.6 | 126.5 | 135.8 |
|  | [36.2] | [32.9] | [36.0] | [36.3] | [34.0] | [31.8] | [34.5] | [34.9] |
| N7 | –276.2 | –292 | –151.7 | –173.6 | –268.9 | –284.8 | (–197.8)^2^ | –206.4 |
| F | –154.5 | –170.2 | –161 | –172.4 | –161.5 | –184.8 | –163.3 | –175.4 |
| C8 | 154.5 | 161.4 | 160.4 | 167.5 | 151.7 | 156.8 | 164.7 | 173.2 |
| C9 | 67.8 | 71.2 | 66.4 | 70.7 | 66.5 | 71.4 | 66.3 | 71.5 |
| C10 | 29.2 | 30.5 | 29.4 | 30.6 | 29.4 | 30 | 29.4 | 30.1 |
| C11 | 28.8 | 28.6 | 29 | 28.6 | 28.9 | 30.2 | 29.2 | 30.5 |
| C12 | 23.2 | 25.1 | 23.2 | 25.4 | 23.5 | 25.1 | 23.6 | 25 |
| C13 | 14.3 | 14.1 | 14.3 | 14.1 | 14.7 | 14 | 14.7 | 14 |
| C14 | 91.2 | 100.9 | 90.1 | 100.7 | 93.3 | 100.7 | 91 | 100.8 |
| C15 | 74.6 | 84.6 | 73.7 | 84.9 | 75.5 | 84.5 | 74.7 | 84.8 |
| C16 | 74.9 | 81.6 | 74.8 | 81.7 | 75.5 | 81.7 | 75.5 | 81.7 |
| C17 | 78.9 | 90.8 | 78.7 | 91 | 80.2 | 90.5 | 80.5 | 91.2 |
| C18 | 18.2 | 20.7 | 18.4 | 20.4 | 18.2 | 20.6 | 18.7 | 20.3 |
| CH3 | 34.7 | 38.7 | 30.4 | 33.7 | – | – | – | – |
| H6 | 7.91 | 8.82 | 7.52 | 8.32 | 7.96 | 8.67 | 7.86 | 8.56 |
| H7 | – | – | – | – | 10.08 | 7.37 | 11.88 | 12.24 |
| H9 | 4.17 | 4.31 | 4.05 | 4.21 | 4.11 | 4.25 | 4.05 | 4.19 |
| H10 | 1.65 | 1.65 | 1.63 | 1.69 | 1.65 | 1.65 | 1.65 | 1.68 |
| H11 | 1.35 | 1.22 | 1.35 | 1.22 | 1.35 | 1.47 | 1.35 | 1.53 |
| H12 | 1.35 | 1.44 | 1.35 | 1.46 | 1.35 | 1.45 | 1.35 | 1.47 |
| H13 | 0.91 | 1.03 | 0.9 | 1.04 | 0.91 | 1.05 | 0.91 | 1.05 |
| H14 | 5.87 | 5.69 | 5.88 | 5.57 | 5.62 | 5.6 | 5.76 | 5.54 |
| H15 | 5.46 | 4.25 | 5.4 | 4.29 | 4.17 | 4.25 | 4.28 | 4.32 |
| H(O15) | – | 5.64 | – | 4.82 | 6.01 | 5.69 | 5.69 | 4.8 |
| H16 | 5.12 | 4.24 | 5.08 | 4.23 | 3.63 | 4.22 | 3.74 | 4.26 |
| H(O16) | – | 3.16 | – | 3.09 | 4.61 | 3.16 | 4.87 | 3.01 |
| H17 | 4.19 | 4.72 | 4.11 | 4.69 | 3.99 | 4.69 | 3.92 | 4.72 |
| H18 | 1.43 | 1.32 | 1.39 | 1.34 | 1.42 | 1.3 | 1.36 | 1.33 |
| H (CH_3_) | 3.29 | 3.29 | 3.27 | 3.36 | – | – | – | – |

^1^ Overlapping signals.

^2^ Linearly extrapolated (not determined experimentally due to overlapping signals), regression details in Table S5.
