# Supplementary material for: Multinuclear NMR Measurements and DFT Calculations for Capecitabine Tautomeric Form Assignment in a Solution
Source: Molecules. 2018 Jan 13;23(1):161. doi: 10.3390/molecules23010161 (PMC6016955; doi:10.3390/molecules23010161)
Supplement: Supplementary file 1 [file molecules-23-00161-s001.zip › TableS8.docx]

**Table S8.** Expanded Table 3: A comparison of the NMR and DFT chemical shifts in ppm and selected spin–spin coupling constants in Hz in square brackets (^1^*J*(C5–F) and ^2^*J*(C4–F)/^2^J(C6–F)) for tautomer **I** of capecitabine in H_2_O and HClO_4_+THF. For comparison, DFT results for hypothetical (undetected in NMR measurements) tautomer **II** in H_2_O are given.

|  | **I in H_2_O** | | **II in H_2_O** | | **I in HClO_4_**+**THF** | |
| --- | --- | --- | --- | --- | --- | --- |
| **Atom** | **NMR** | **DFT** | **NMR** | **DFT** | **NMR** | **DFT** |
| N1 | –225 | –226.7 | – | –243.9 | –221 | –218.4 |
| C2 | 157 | 164.9 | – | 158.1 | 145.6 | 153.2 |
| N3 | (–*158.4*)^1^ | –165.4 | – | –244.3 | –225.8 | –250.3 |
| C4 | 157.1 | 163.2 | – | 164.3 | 152.9 | 159.9 |
|  | [12.6] | [8.0] | – | [16.6] | [23.8] | [16.2] |
| C5 | 140.5 | 145.6 | – | 149 | 136.4 | 141.1 |
|  | [245.2] | [266.6] | – | [257.1] | [233.4] | [264.0] |
| C6 | 131.3 | 140.6 | – | 138.3 | 135.5 | 150.1 |
|  | [33.4] | [32.9] | – | [36.3] | [33.0] | [30.2] |
| N7 | (–*271.6*)^1^ | –283.3 | – | –214.5 | –259.4 | –282.4 |
| F | –163.2 | –182.2 | – | –177.1 | –165.8 | –181.9 |
| C8 | 155.6 | 160.6 | – | 174.4 | 154.3 | 160.7 |
| C9 | 70 | 72.9 | – | 72.2 | 69.2 | 77.7 |
| C10 | 30.3 | 29.5 | – | 29.7 | 28.9 | 29.6 |
| C11 | 30 | 30.2 | – | 30.5 | 28.6 | 30.3 |
| C12 | 24.4 | 24.8 | – | 24.9 | 23.1 | 24.8 |
| C13 | 16 | 13.8 | – | 13.8 | 14.3 | 13.8 |
| C14 | 94.6 | 101 | – | 101 | 94.5 | 104.1 |
| C15 | 77.4 | 84.5 | – | 84.8 | 75.5 | 84.1 |
| C16 | 76.9 | 82.4 | – | 82.2 | 75.1 | 81.9 |
| C17 | 82.5 | 91.1 | – | 91.6 | 81.3 | 92.6 |
| C18 | 20.1 | 20.2 | – | 19.8 | 18.1 | 20.1 |
| H6 | 8.06 | 8.89 | – | 8.78 | 8.36 | 9.43 |
| H7 | n.a.^2^ | 7.79 | – | – | 10.08 | 8.09 |
| H9 | 4.23 | 4.36 | – | 4.27 | 4.32 | 4.64 |
| H10 | 1.7 | 1.77 | – | 1.72 | 1.73 | 1.82 |
| H11 | 1.35 | 1.48 | – | 1.51 | 1.38 | 1.51 |
| H12 | 1.34 | 1.45 | – | 1.47 | 1.36 | 1.49 |
| H13 | 0.88 | 1.05 | – | 1.06 | 0.9 | 1.08 |
| H14 | 5.78 | 5.81 | – | 5.75 | 5.69 | 5.76 |
| H15 | 4.3 | 4.49 | – | 4.54 | 4.26 | 4.45 |
| H(O15) | n.a.^2^ | 5.59 | – | 4.83 | 6.01 | 4.17 |
| H16 | 3.88 | 4.39 | – | 4.4 | 3.81 | 4.4 |
| H(O16) | n.a.^2^ | 3.48 | – | 3.44 | 4.61 | 2.95 |
| H17 | 4.21 | 4.77 | – | 4.77 | 4.05 | 4.88 |
| H18 | 1.47 | 1.41 | – | 1.39 | 1.42 | 1.34 |

^1^ Linearly extrapolated (not determined experimentally), regression details in Table S5.

^2^ Not available.
