# Supplementary material for: Multinuclear NMR Measurements and DFT Calculations for Capecitabine Tautomeric Form Assignment in a Solution
Source: Molecules. 2018 Jan 13;23(1):161. doi: 10.3390/molecules23010161 (PMC6016955; doi:10.3390/molecules23010161)
Supplement: Supplementary file 1 [file molecules-23-00161-s001.zip › TableS9.docx]

**Table S9.** Input data for linear regression of Table S5: Experimental NMR and theoretical DFT ^15^N chemical shifts discussed in the present work. The selected hydrogens are located in the central region of the capecitabine molecule.

| No**.** | **Molecule** | **Atom** | **NMR** | **DFT** |
| --- | --- | --- | --- | --- |
| 1 | **I** (THF) | N1 | –221.7 | –229.1 |
| 2 | **I** (THF) | N3 | –139.3 | –159.6 |
| 3 | **I** (THF) | N7 | –268.9 | –284.8 |
| 4 | **II** (THF) | N1 | –244.6 | –248.1 |
| 5 | **II** (THF) | N3 | –236.5 | –246.5 |
| 6 | **II** (THF) | N7 | (*–197.8*)^1^ | –206.4 |
| 7 | **I** (H_2_O) | N1 | –225.0 | –226.7 |
| 8 | **I** (H_2_O) | N3 | (*–158.4*)^1^ | –165.4 |
| 9 | **I** (H_2_O) | N7 | (*–271.6*)^1^ | –283.3 |
| 10 | **I** (HClO_4_+THF) | N1 | –221.0 | –218.4 |
| 11 | **I** (HClO_4_+THF) | N3 | –225.8 | –250.3 |
| 12 | **I** (HClO_4_+THF) | N7 | –259.4 | –282.4 |
| 13 | **2** (THF) | N1 | –226.2 | –218.4 |
| 14 | **2** (THF) | N3 | –123.9 | –124.5 |
| 15 | **2** (THF) | N7 | –276.2 | –292.0 |
| 16 | **3** (THF) | N1 | –255.9 | –255.8 |
| 17 | **3** (THF) | N3 | –245.6 | –255.8 |
| 18 | **3** (THF) | N7 | –151.7 | –173.6 |

^1^ Based on the linear regression of NMR (*y*) vs. DFT (*x*): *y = a + b·x*, where *a* = 0.2996, *b* = 0.9597, see Table S5.
