# Supplementary figures and images for: Multinuclear NMR Measurements and DFT Calculations for Capecitabine Tautomeric Form Assignment in a Solution
Source: Molecules. 2018 Jan 13;23(1):161. doi: 10.3390/molecules23010161 (PMC6016955; doi:10.3390/molecules23010161)

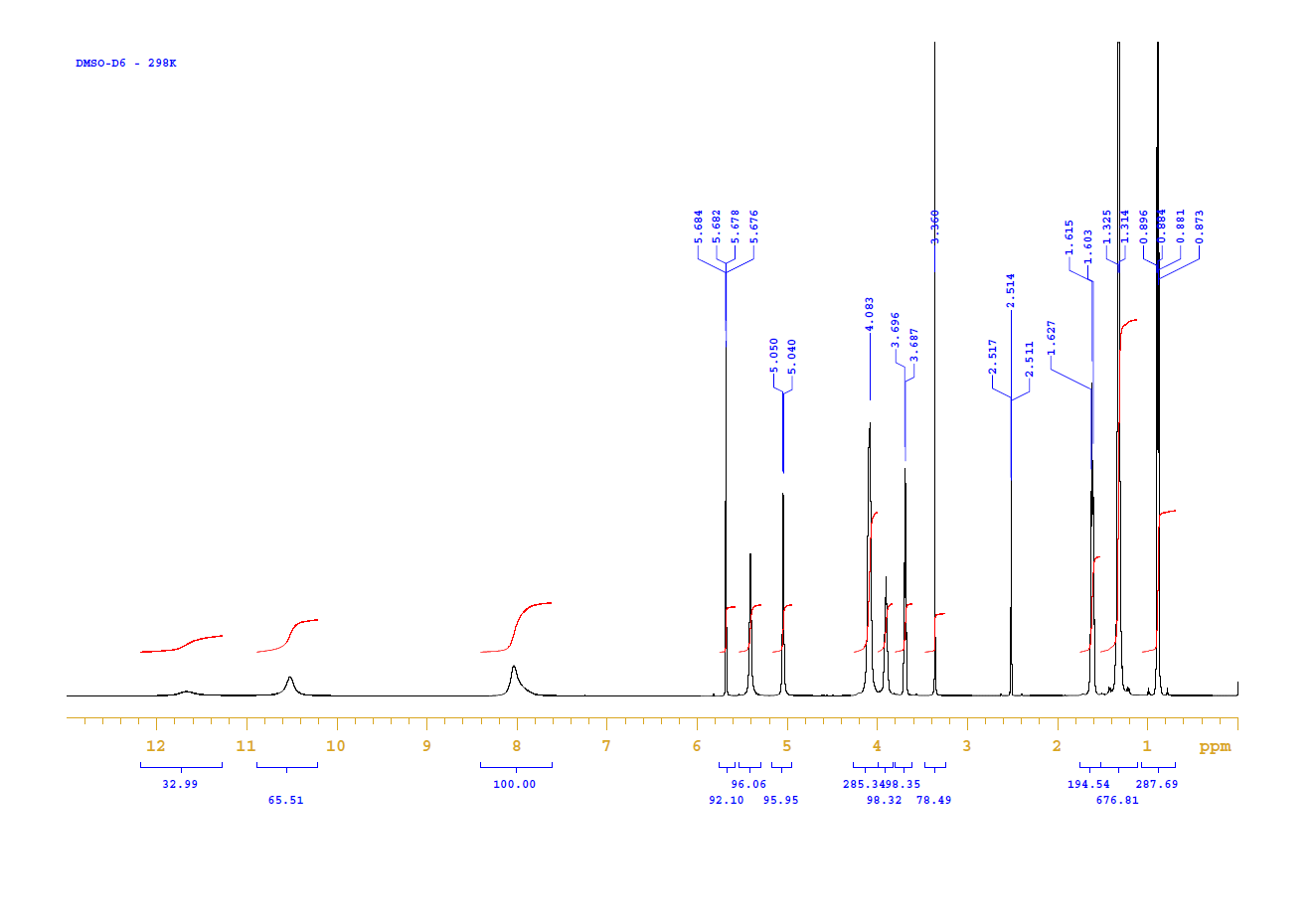

Supplement: Supplementary file 1 [file molecules-23-00161-s001.zip › Fig_S2.tif]

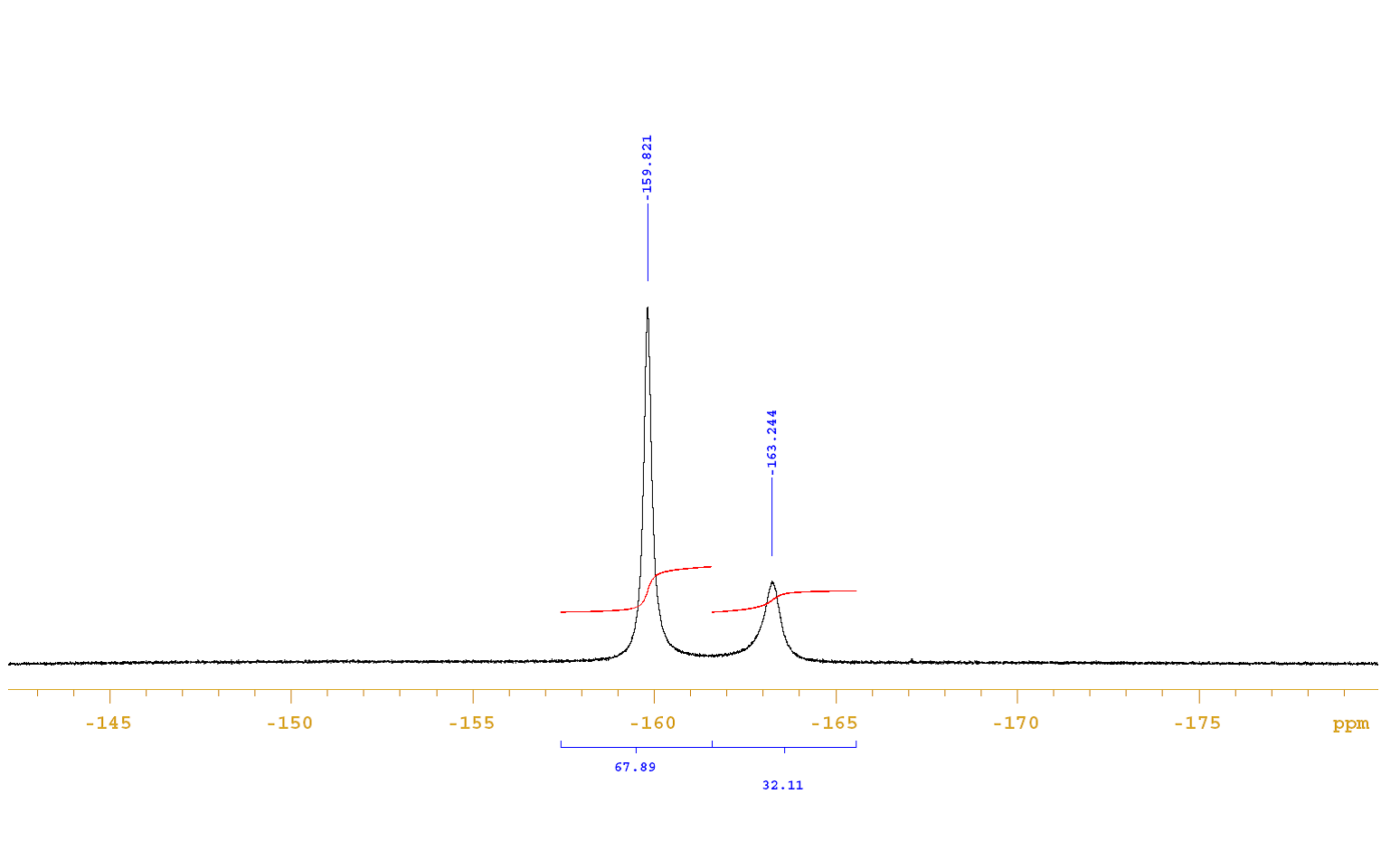

Supplement: Supplementary file 1 [file molecules-23-00161-s001.zip › Fig_S3.tif]

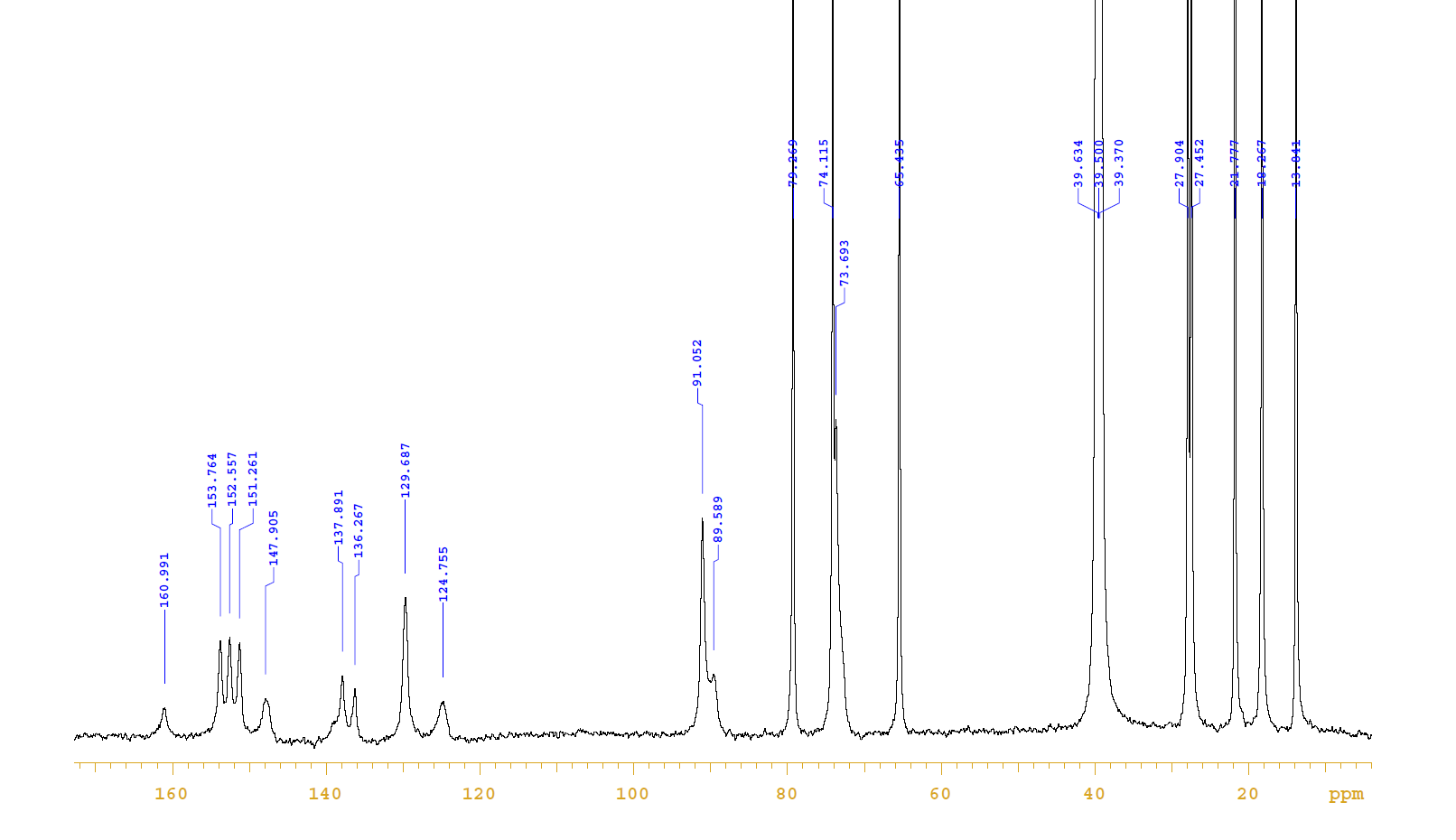

Supplement: Supplementary file 1 [file molecules-23-00161-s001.zip › Fig_S4.tif]

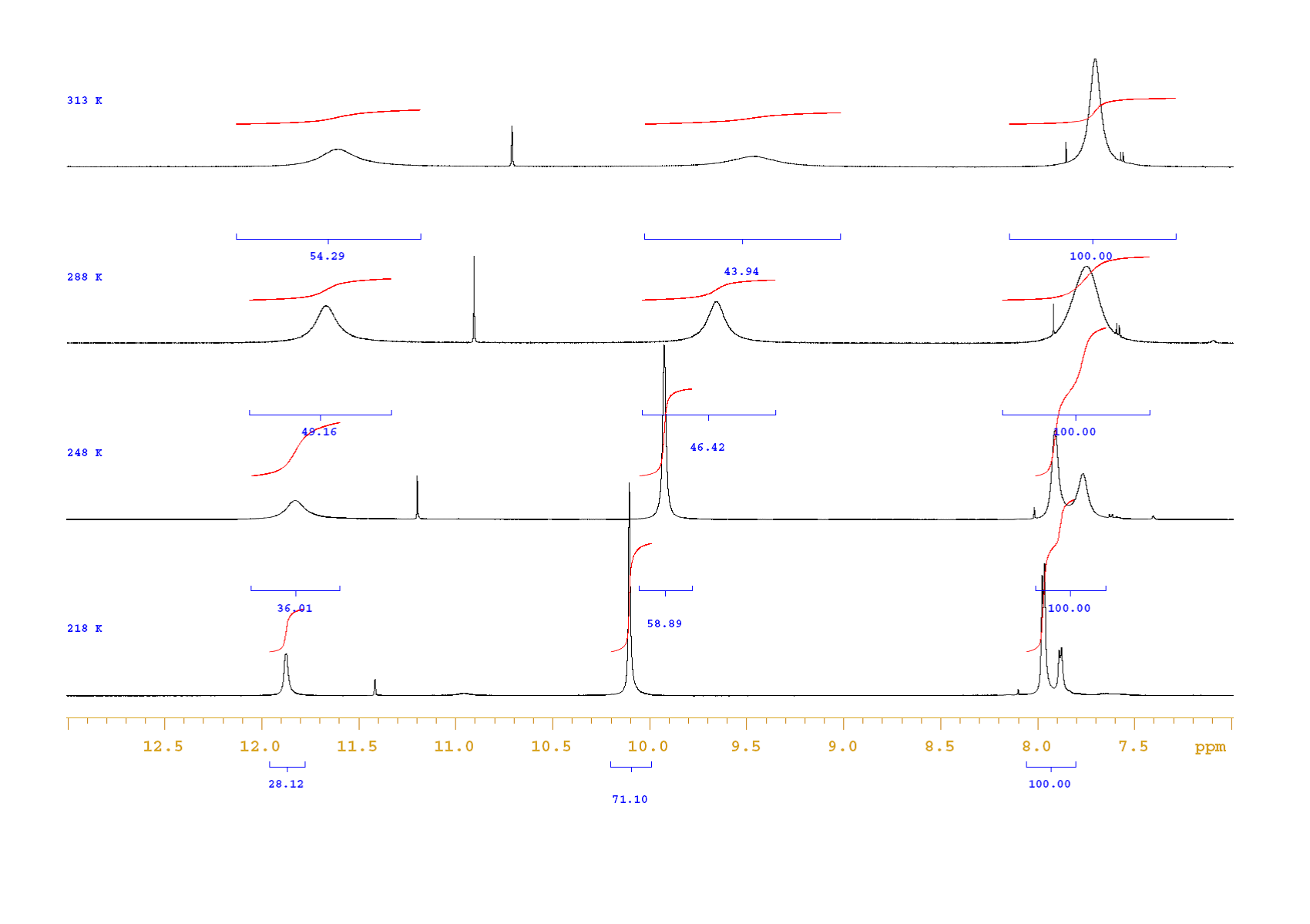

Supplement: Supplementary file 1 [file molecules-23-00161-s001.zip › Fig_S5.tif]

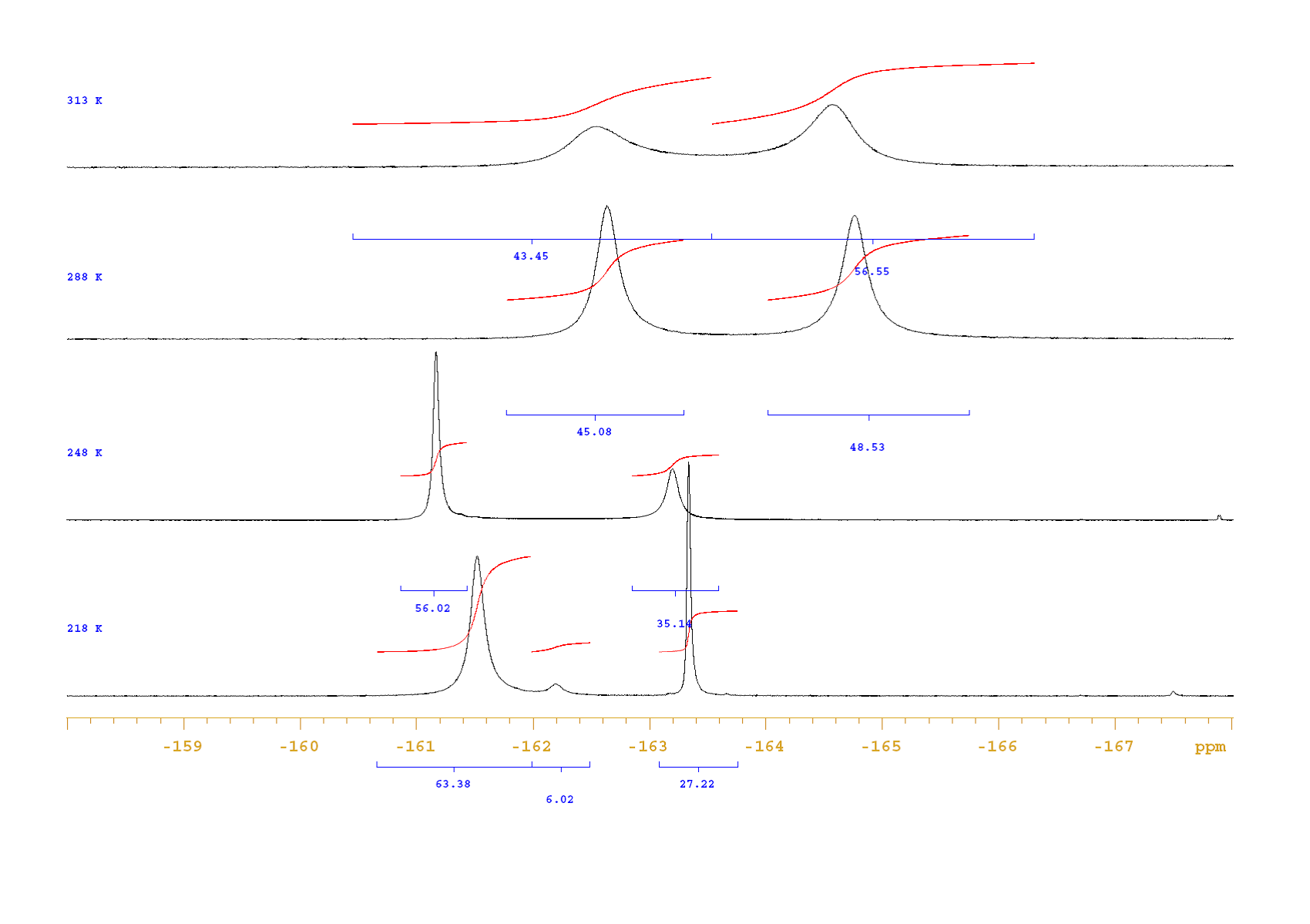

Supplement: Supplementary file 1 [file molecules-23-00161-s001.zip › Fig_S6.tif]

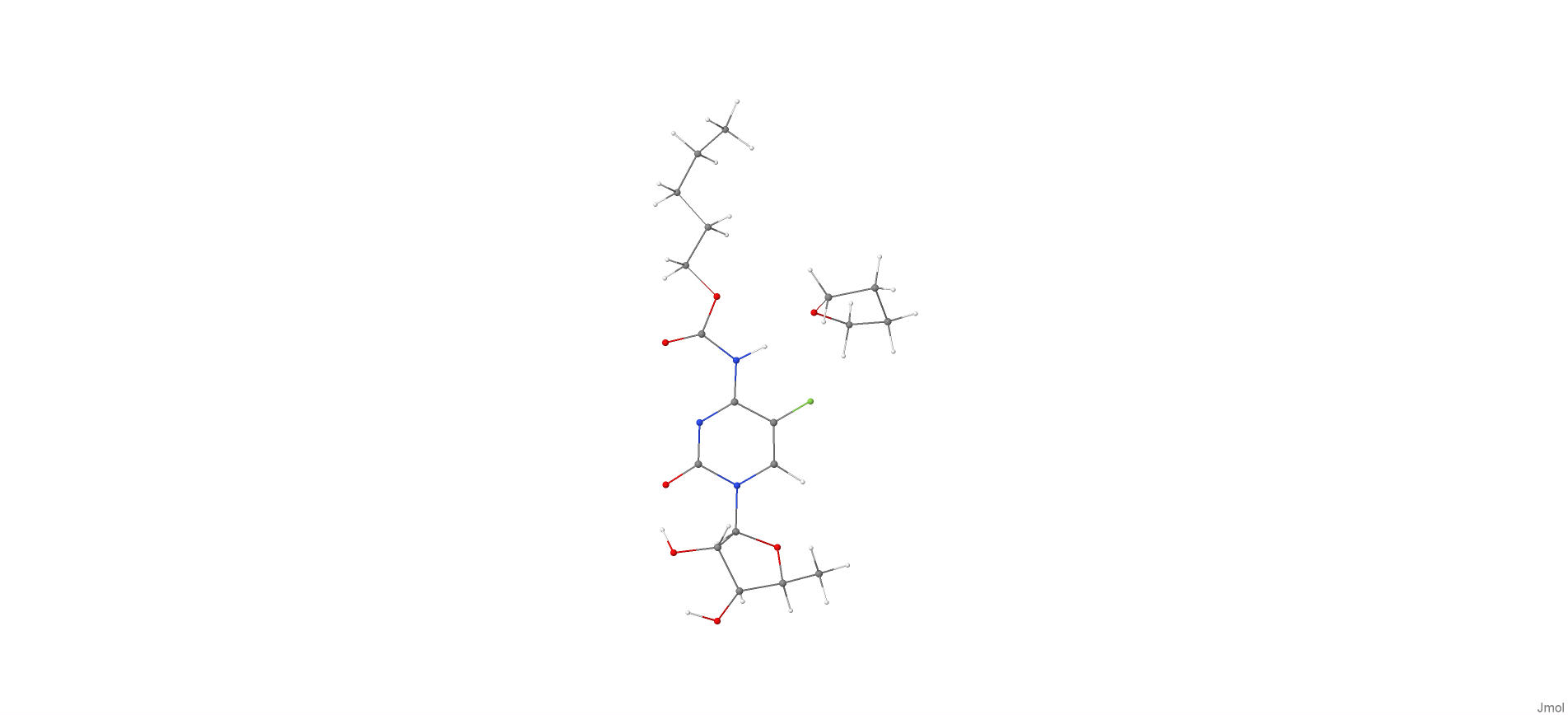

Supplement: Supplementary file 1 [file molecules-23-00161-s001.zip › Fig_S7a.jpg]

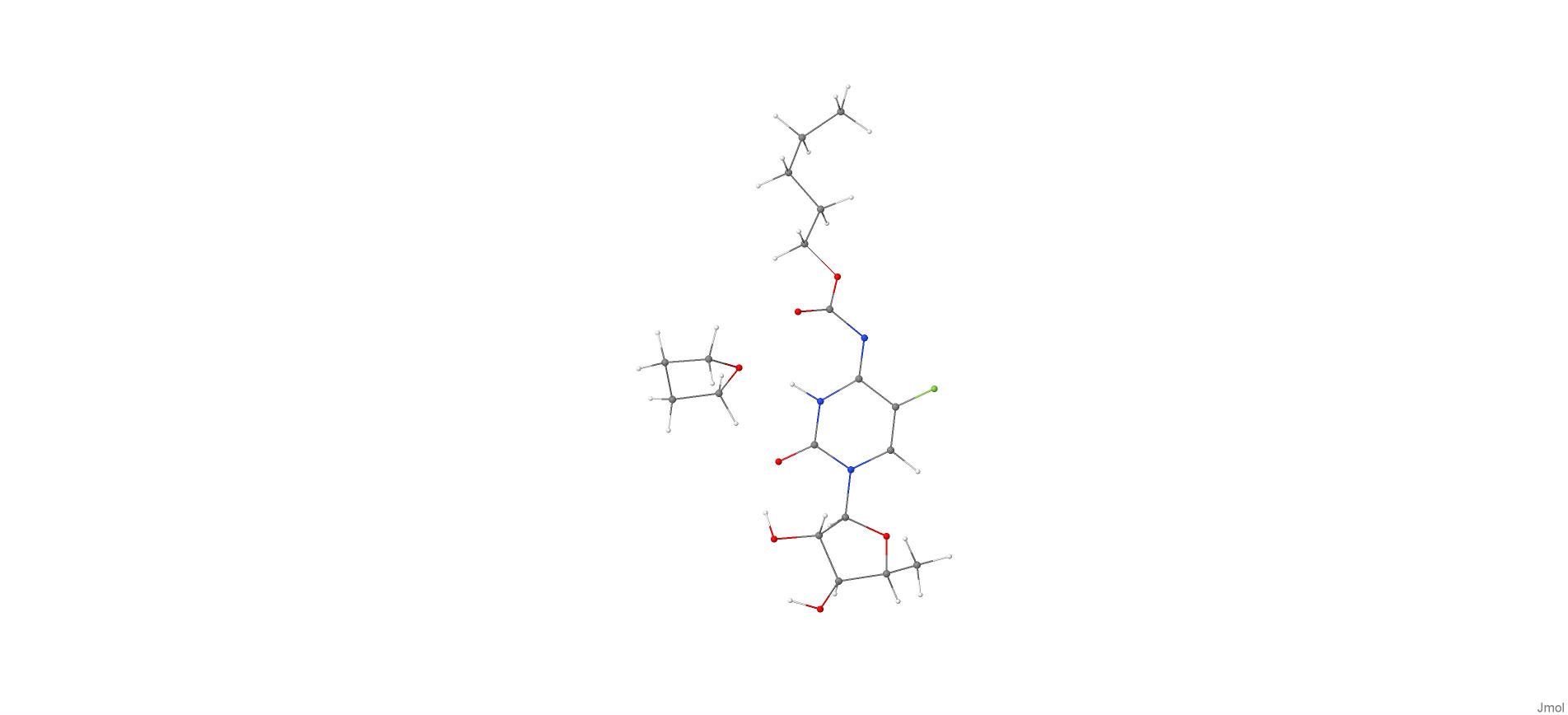

Supplement: Supplementary file 1 [file molecules-23-00161-s001.zip › Fig_S7b.jpg]

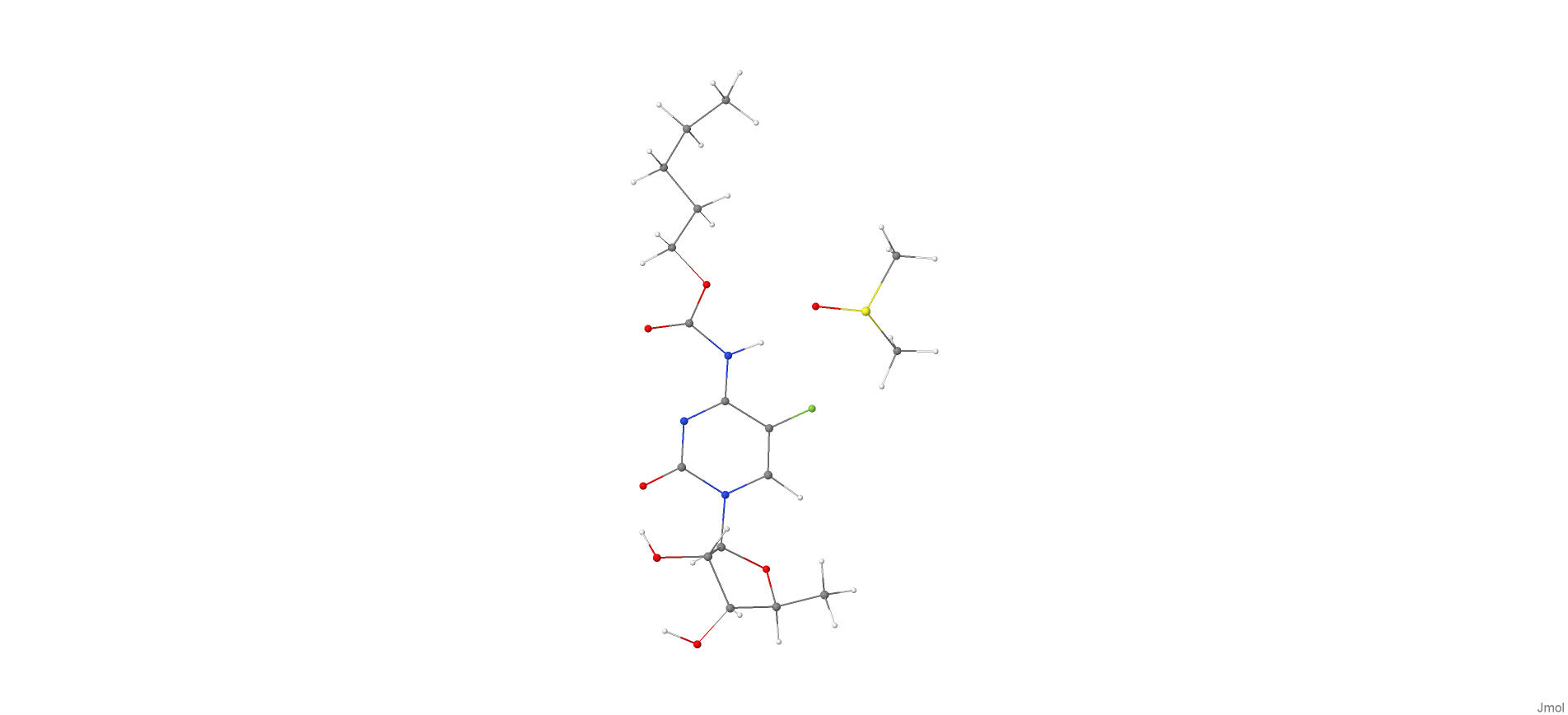

Supplement: Supplementary file 1 [file molecules-23-00161-s001.zip › Fig_S7c.jpg]

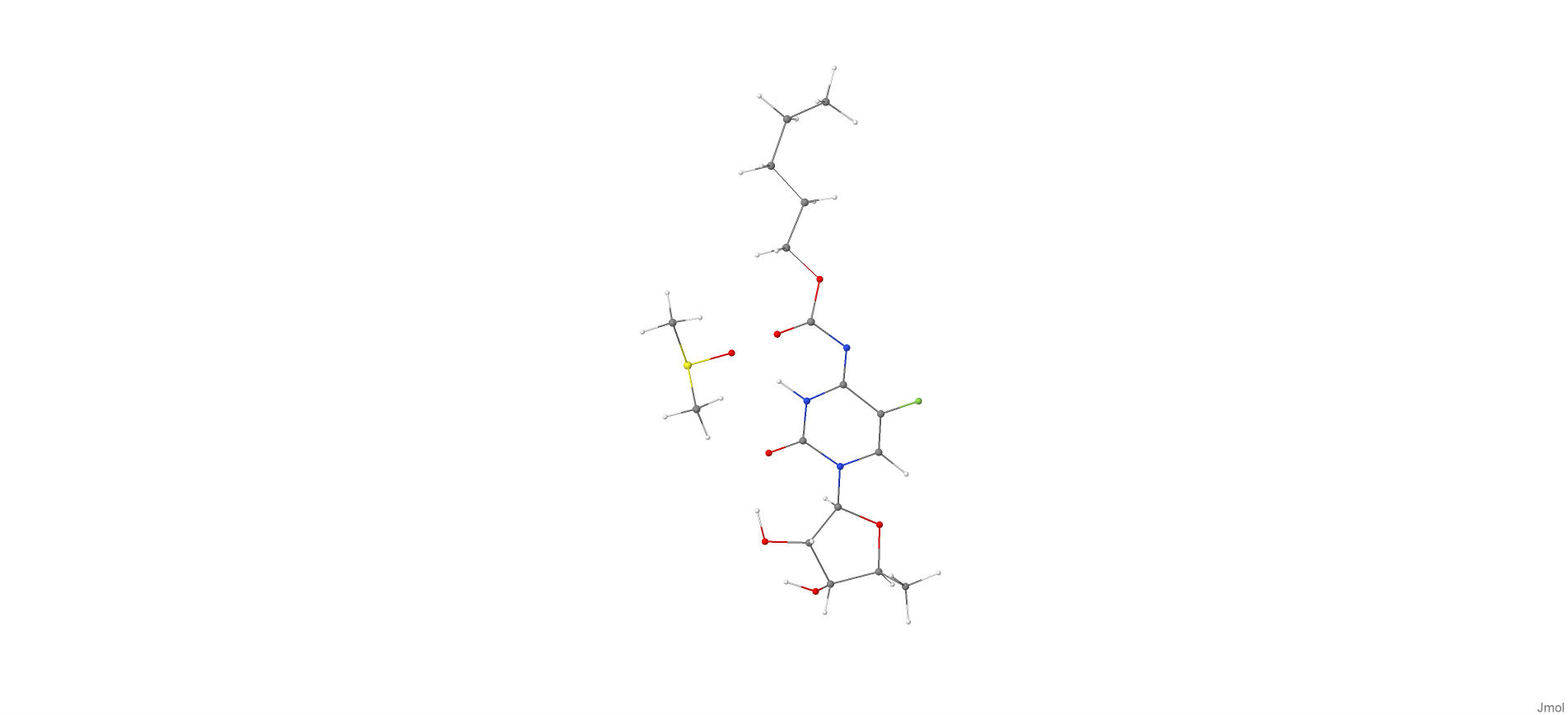

Supplement: Supplementary file 1 [file molecules-23-00161-s001.zip › Fig_S7d.jpg]

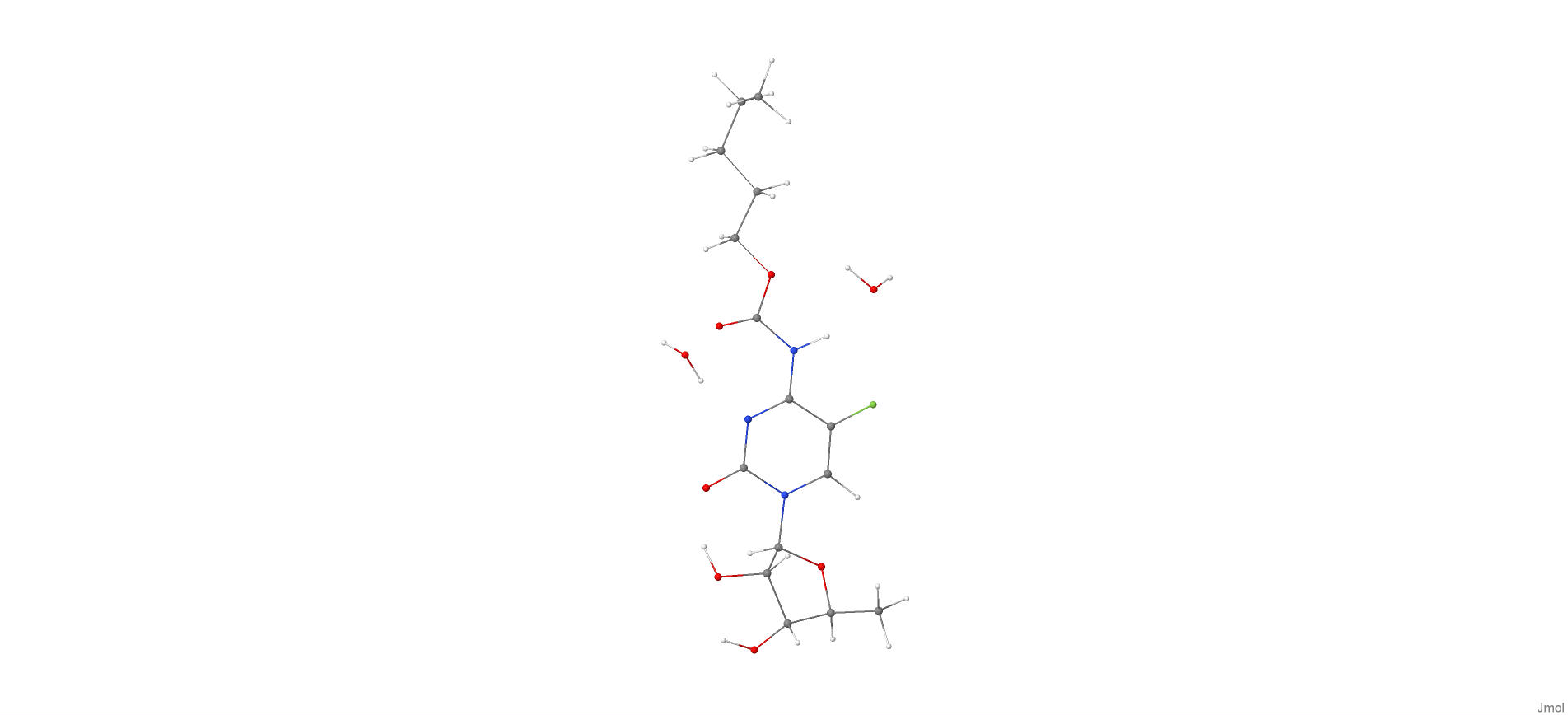

Supplement: Supplementary file 1 [file molecules-23-00161-s001.zip › Fig_S7e.jpg]

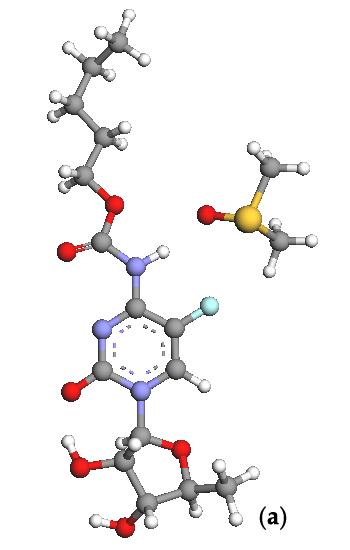

Supplement: Supplementary file 1 [file molecules-23-00161-s001.zip › Fig_S7_a.tiff]

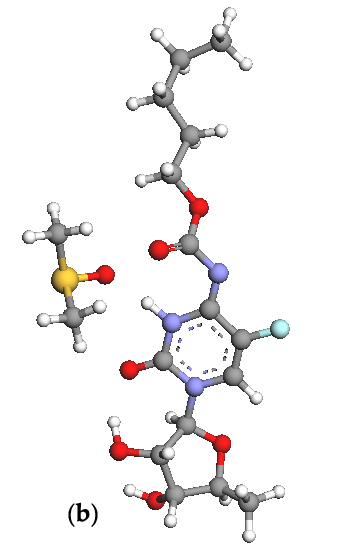

Supplement: Supplementary file 1 [file molecules-23-00161-s001.zip › Fig_S7_b.tiff]

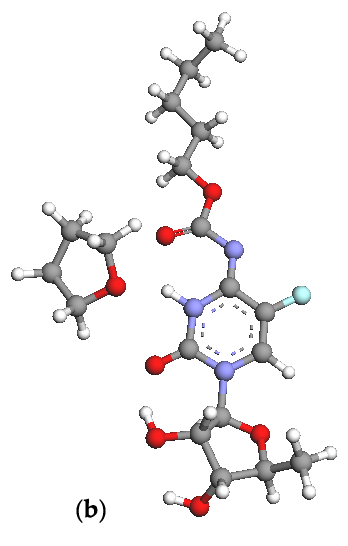

Supplement: Supplementary file 1 [file molecules-23-00161-s001.zip › Fig_S8_a.tiff]

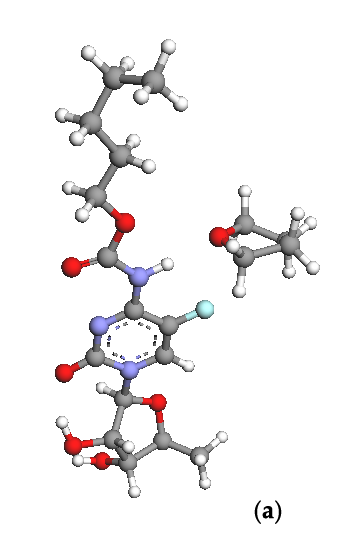

Supplement: Supplementary file 1 [file molecules-23-00161-s001.zip › Fig_S8_b.tiff]

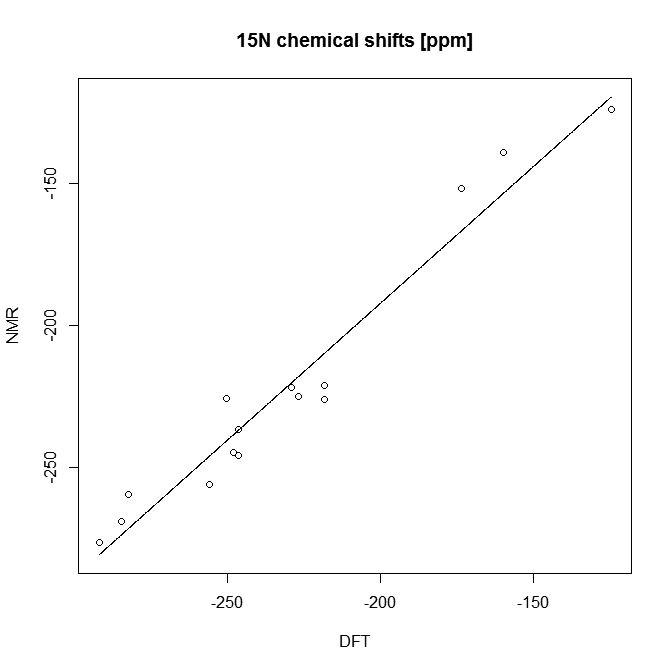

Supplement: Supplementary file 1 [file molecules-23-00161-s001.zip › Fig_S9.tiff]
